# Transcriptomic profiling of *Bacillus amyloliquefaciens* FZB42 in response to maize root exudates

**DOI:** 10.1186/1471-2180-12-116

**Published:** 2012-06-21

**Authors:** Ben Fan, Lilia C Carvalhais, Anke Becker, Dmitri Fedoseyenko, Nicolaus von Wirén, Rainer Borriss

**Affiliations:** 1 Institute of Forest Protection, Nanjing Forestry University, Longpan Road 159, 210037, Nanjing, China; 2 Institut für Biologie Bakteriengenetik, Humboldt Universität Berlin, Chausseestrasse 117, D-10115, Berlin, Germany; 3 Molekulare Genetik, Institut für Biologie III, Albert-Ludwigs-Universität Freiburg, Schänzlestrasse 1, D-79104, Freiburg, Germany; 4 Leibniz Institute for Plant Genetics and Crop Plant Research, Corrensstr. 3, 06466, Gatersleben, Germany; 5 ABiTEP GmbH, Glienicker Weg 185, D-12489, Berlin, Germany

## Abstract

**Background:**

Plant root exudates have been shown to play an important role in mediating interactions between plant growth-promoting rhizobacteria (PGPR) and their host plants. Most investigations were performed on Gram-negative rhizobacteria, while much less is known about Gram-positive rhizobacteria. To elucidate early responses of PGPR to root exudates, we investigated changes in the transcriptome of a Gram-positive PGPR to plant root exudates.

**Results:**

*Bacillus amyloliquefaciens* FZB42 is a well-studied Gram-positive PGPR. To obtain a comprehensive overview of FZB42 gene expression in response to maize root exudates, microarray experiments were performed. A total of 302 genes representing 8.2% of the FZB42 transcriptome showed significantly altered expression levels in the presence of root exudates. The majority of the genes (261) was up-regulated after incubation of FZB42 with root exudates, whereas only 41 genes were down-regulated. Several groups of the genes which were strongly induced by the root exudates are involved in metabolic pathways relating to nutrient utilization, bacterial chemotaxis and motility, and non-ribosomal synthesis of antimicrobial peptides and polyketides.

**Conclusions:**

Here we present a transcriptome analysis of the root-colonizing bacterium *Bacillus amyloliquefaciens* FZB42 in response to maize root exudates. The 302 genes identified as being differentially transcribed are proposed to be involved in interactions of Gram-positive bacteria with plants.

## Background

Plant growth-promoting rhizobacteria (PGPR) are generally referred to as a heterogeneous group of bacteria which colonize the rhizoplane and/or rhizosphere and stimulate plant growth [1,2]. PGPR have been commercially exploited as biofertilizers to improve the yield of crops. Some PGPR have also been successfully used as biocontrol agents to prevent plant diseases caused by phytopathogens, especially some soil-borne diseases [3-5]. The investigations on the interactions between PGPR and their host plants can not only contribute to our understanding of eukaryote-prokaryote relationships, but also have fundamental implications for designing new strategies to promote agricultural plant production.

In recent years, there is increasing evidence that plant root exudates play a key role in plant-microbe interactions [6-10]. Root exudates consist of an enormous range of compounds, among which some can attract beneficial associative bacteria to overcome stress situations [8]. On the other hand, root exudates contain low molecular-weight carbon such as sugars and organic acids that primarily act as energy sources for rhizobacteria and shape bacterial communities in the rhizosphere [11]. To date, however, it remains unclear how root exudates exert differential effects on rhizobacteria and which mechanisms or pathways make rhizobacteria responsive to plant root exudates.

Transcriptome analyses are an efficient approach to study host-microbe interactions at a wider scale. So far, the use of this approach to analyse bacterial gene expression has been extensively used to study pathogenic microbes infecting their host [12]. Only a few studies were performed with beneficial PGPR [13-15]. Several genes from *Pseudomonas aeruginosa* involved in metabolism, chemotaxis and type II secretion were identified to respond to sugar-beet root exudates [13]. In another study, it has been suggested that the availability of particular metabolites in root exudates, especially amino acids and aromatic compounds, support *Pseudomonas putida* to colonize the rhizosphere [14]. *Rhizobium leguminosarum* was grown in the rhizospheres of its host-legume pea and two other non-host plants, alfalfa and sugar-beet. Although numerous sugar and putative complex carbohydrate transport systems are induced in the rhizosphere, they are less important carbon sources than organic acids. A common core of rhizosphere-induced genes was identified [15].

So far, studies on the impact of root exudates on PGPR, have been conducted with Gram-negative bacteria, mainly *Azospirillum* and *Pseudomonas* spp. [16,17]. Related studies performed with Gram-positive PGPR are still missing. Owing to differences in lifestyle and physiology, Gram-positive and Gram-negative rhizobacteria may use distinct mechanisms when interacting with plants. Due to their ability to produce durable endo-spores, bacilli are now preferred in manufacturing biofertilizer formulations [18], however, their successful application is still hampered by a lack of knowledge about factors determining interactions between plants and those bacteria, especially root colonization is far from being completely understood.

*Bacillus amyloliquefaciens* FZB42 is a plant root-colonizing Gram-positive PGPR. A series of studies has elucidated several aspects of this rhizobacterium, particularly the molecular basis of its plant growth-promoting activity, which is mainly based on the production of secondary metabolites suppressing competitive microbial pathogens occurring in the plant rhizosphere, the secretion of the plant growth hormone auxin, and the synthesis of volatiles stimulating plant growth and induced systemic resistance (ISR) [19-21]. In the case of Gram-positive PGPR, however, it is still not clear how they maneuver their gene expression when exposed to plant-derived compounds. To address this question, the commercially established FZB42 wild type strain from *Bacillus amyloliqufaciens* was tested in this study for its transcriptomic responses to maize root exudates using a two-color DNA microarray system.

## Results and discussion

### Composition of maize root exudates

Maize root exudates were collected from axenic hydroponic cultures and analysed by HPLC for organic acids, amino acids, and oligosaccharides, which have been previously reported to be among the major ingredients in root exudates [8,22-24].

**Figure 1:**
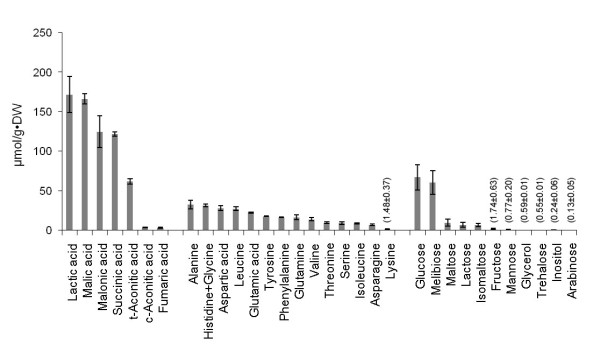
** Composition and concentration of the maize root exudates.** Exudates collected from the roots of maize seedlings were assayed by HPLC. Organic acids, amino acids, and carbohydrates were quantified and those which had a concentration of >0.1 μmol g-1(dry weight) were included in the graph. Proline, a known constituent of maize root exudate, was not detected since the derivatization reagent (OPA) used reacts only with primary amino groups.

Among the compounds detected, in particular organic acids such as malic acid, malonic acid, succinic acid and trans-aconitic acid, were present at highest concentrations (Figure 1). Corroborating an earlier report [25], we found that lactic acid was a main constituent of maize root exudates. A variety of amino acids was also detected. Glucose and melibiose were the most prominent sugars occurring in root exudates. According to this analysis, most low-molecular weight organic carbon appeared to be present in the form of organic acids.

### Overall changes in gene expression in response to root exudates

**Figure 2:**
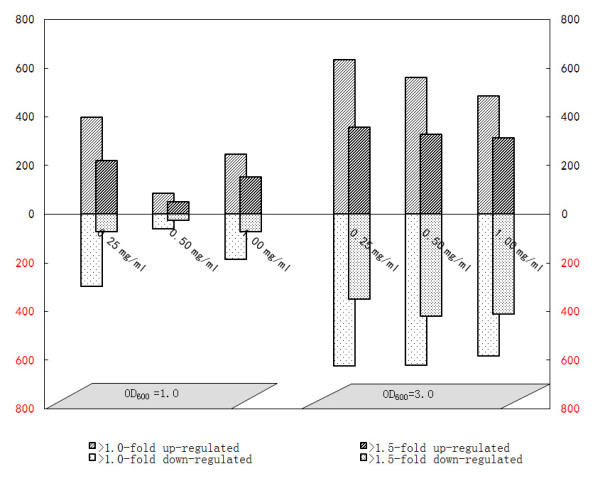
** Number of FZB42 genes altered in transcription in response to root exudates at different exudate concentrations and cell densities.** Maize root exudates were supplemented in three concentrations (0.25 mg/ml, 0.5 mg/ml and 1.0 mg/ml) to FZB42 cultures and total RNA was prepared from the bacterial cells harvested at two optical densities (OD_600_ = 1.0 and OD_600_ = 3.0). Genes significantly altered in transcription (q ≤ 0.01 and fold change ≥1.5) by presence of root exudates are represented in the figure.

In the rhizosphere, root exudates may occur at high concentrations in certain microenvironments, e.g. in vicinity of root tips [26], but their concentration in specific niches of the environment is unknown. Therefore, the choice of a physiologically relevant concentration of exudates to be used for microarray experiments can only be tentative. Based on a previous study on changes in the proteomics of FZB42 [27], three exudate concentrations (0.25 g l^-1^, 0.5 g l^-1^ and 1.0 g l^-1^) were applied to liquid cultures of FZB42, and bacterial cells were harvested for RNA extraction at two growth stages (OD_600_ = 1.0 and OD_600_ = 3.0). For simplicity, the two population densities were referred to as OD1.0 and OD3.0 throughout this paper, respectively. A concentration of 0.25 g l^-1^ was sufficient to result in a significant response of FZB42 transcriptome. When bacteria were cultured at OD3.0 the number of up-regulated genes gradually decreased with increasing root exudate concentration, suggesting that some compounds need to occur at lower abundance to induce gene expression, or that gene transcription in general may be suppressed at high concentrations of some exudates components (Figure 2). More transcripts were significantly altered (q ≤ 0.01) at the transition to the stationary growth (OD 3.0) than at the exponential growth (OD1.0) (Figure 2), suggesting that OD 3.0 was a sampling point which reflected more clearly the effect of root exudates on FZB42 than OD1.0. For these reasons, the exudate concentration of 0.25 g l^-1^ and the OD3.0 for harvesting of cells were used for all subsequent microarray experiments.

**Figure 3:**
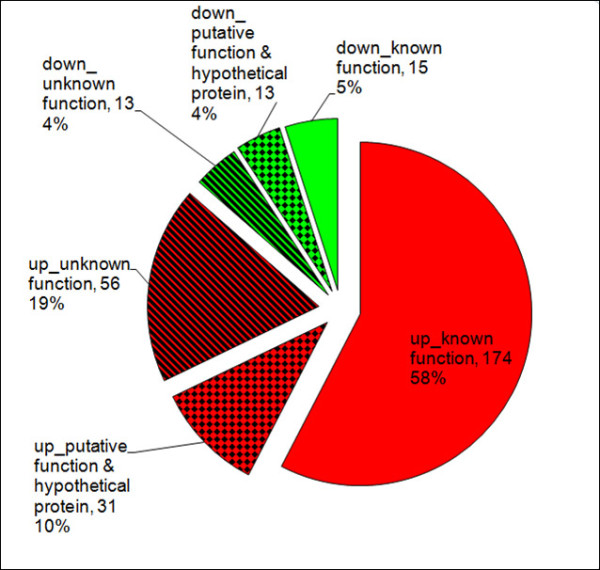
** Overview of groups of the 302 genes altered in transcription by root exudates.** A total of 302 genes were significantly altered (q ≤ 0.01 and fold change ≥1.5) in transcription by the maize root exudates. “Up” indicates genes that were up-regulated in presence root exudates, while “down” the ones that were down-regulated by the root exudates. The genes encoding a product with known or unknown function and those encoding a hypothetical protein were indicated. The number of genes of each section and their percentage is depicted.

**Figure 4:**
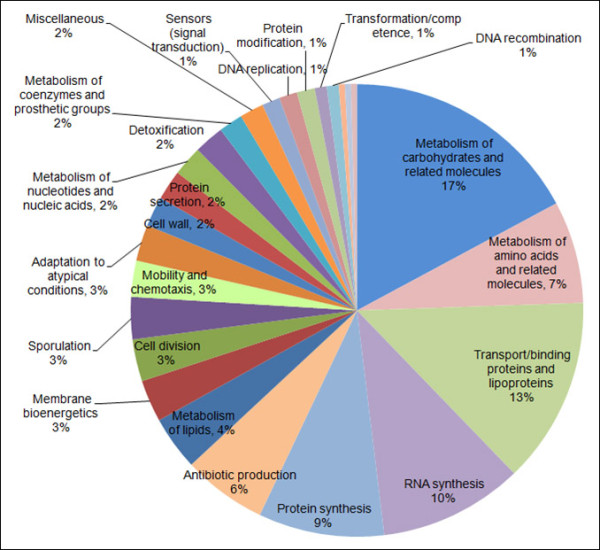
** Distribution in various functional categories of the genes altered in transcription by root exudates.** Among the 302 genes altered in transcription by maize root exudates at OD3.0, those with known (189 genes) or putative (44 genes) products were classified according to their function. The percentage of each group is indicated.

Six independent experiments were performed and the genes whose transcription fulfilled the condition of yielding a q value not greater than 0.01 (q ≤ 0.01) and a fold change not less than 1.5 (FCH ≥ 1.5) were regarded as being significantly influenced by root exudates. A total of 302 genes, representing 8.2% of the FZB42 transcriptome, were significantly regulated in their transcript levels by the applied root exudates (see supplemental material Additional file 1: Tables S1, S2, and S3). The majority of these genes (261 genes) was up-regulated, whereas only 41 genes were down-regulated (Figure 3). Although most of the regulated genes have been functionally annotated, a significant proportion (~23%) remained of unknown function, among which 19 genes were unique for FZB42. In addition, 44 genes (~15%) encoded either hypothetical proteins or proteins with putative functions (Figure 3). The distribution in various functional categories of all the gene with known (189 genes) or putative (44 genes) products are summarized in Figure 4.

### Validation of microarray result by real-time PCR

**Figure 5:**
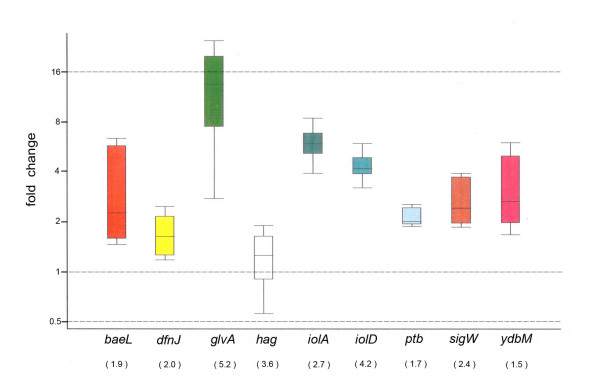
** Fold-change of differentially expressed genes selected for validation by Real-time PCR.** The fold changes revealed by real-time PCR of the selected genes were determined using the software REST. Three repeats were performed for each gene. For comparison, the fold changes obtained in microarray analysis were shown in parenthesis below each specific gene. The boxes represent the distance between the 25th and the 75th percentile. The lines in the boxes represent the median gene expression. Whiskers represent the minimum and maximum observations.

Nine up-regulated genes with different levels of fold changes in expression (1.5 ~ 5.2 fold) were chosen to be evaluated by quantitative real-time PCR. All these genes were confirmed to be significantly up-regulated in the presence of root exudates (Figure 5). The fold change of each gene revealed by real-time PCR was similar to that obtained in the microarray experiments (Figure 5). In summary, the real-time PCR suggested that the microarray data were reliable.

### The regulated genes with known function

Among the 302 genes with significantly altered expression by root exudates, 189 were annotated with known functions. These were categorized into various classes [28], such as cell envelope and cellular processes, intermediary metabolism, information pathway and other functions . In these categories, three groups (Table 1) contained the largest numbers of genes and at least one third of the genes within these groups had a fold change of ≥2.0. This suggests that these three groups of genes were strongly affected by root exudates:

**Table 1:** Functional categories* of the FZB42 genes significantly regulated by the maize root exudates and with known functions

**Classification code_Functional category**	**Nr. of the genes included**
1_cell envelope and cellular processes	58
1.7_ Cell division	6
1.1_ Cell wall	5
1.4_ Membrane bioenergetics	7
**1.5_ Mobility and chemotaxis**	**6**
**1.3_ Sensors (signal transduction)**	**2**
1.6_ Protein secretion	5
1.8_ Sporulation	7
1.1_ Transformation/competence	2
1.2_ Transport/binding proteins and lipoproteins	18
2_intermediary metabolism	59
**2.1_Metabolism of carbohydrates and related molecules**	**34**
**2.2_ Metabolism of amino acids and related molecules**	**12**
2.5_ Metabolism of coenzymes and prosthetic groups	4
2.4_ Metabolism of lipids	5
2.3_ Metabolism of nucleotides and nucleic acids	4
3_information pathways	45
3.3_ DNA recombination	1
3.1_ DNA replication	3
3.8_ Protein modification	2
3.7_ Protein synthesis	20
3.6_ RNA modification	1
3.5_ RNA synthesis	18
4_other functions	27
4.1_ Adaptation to atypical conditions	6
4.2_ Detoxification	4
4.6_ Miscellaneous	3
4.4_ Phage-related functions	1
**4.3_ Antibiotic production**	**13**
In total	189

The categories in which more than one third of the genes had a fold change of ≥2.0 were highlighted in bold text (Refer to experiment “Response to RE”: E-MEXP-3421). *The genes were categorized according to [28].

**Figure 6:**
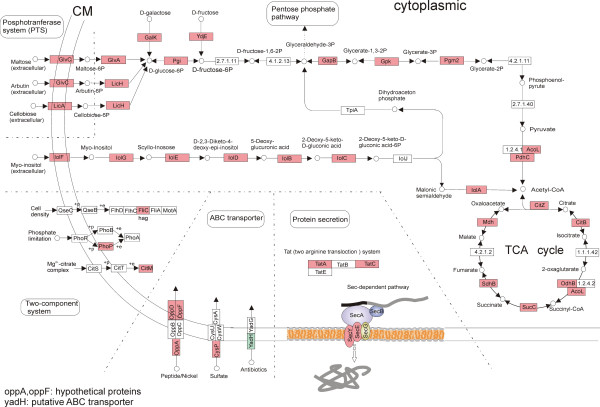
**A subset of the up-regulated genes with known function in response to maize root exudates.** The significantly up-regulated genes by the root exudates were mapped in the KEGG pathway and the diagram was accordingly adapted. The products encoded by the up-regulated genes were highlighted in red, whilst the down-regulated YadH was highlighted in green. CM stands for cell membrane.

i) The transcription of 46 genes involved in carbon and nitrogen utilization was altered in response to root exudates, with 43 of them being up-regulated. These 46 genes were involved in different aspects of the metabolism of carbohydrates, amino acids and related metabolites. To obtain a more comprehensive understanding of their relevance in the metabolic context, the genes were mapped into the KEGG pathway and a representation of metabolic pathways was constructed (Figure 6). A total of 12 genes encoding enzymes involved in the Embden-Meyerhof-Parnas (EMP) pathway (including *pgi* encoding for glucose-6-phosphate isomerase) and the TCA cycle were significantly up-regulated. These genes covered almost the entire glycolysis and TCA pathway. Nearly a quarter of the genes with altered transcription (46 out of 189) were involved in uptake or utilization of nutrients. This observation corroborated that root exudates serve as energy sources in the interaction between roots and rhizobacteria.

Among the up-regulated genes, *glvAglv*C and *glvR* showed the highest fold change (*glvA*: 5.2-fold up-regulated, *glvC*: 2.5-fold up-regulated, *glvR*: 4.4-fold up-regulated). The enhancement of *glvA* expression was also validated by real-time PCR as well as by the proteomic data (unpublished). The three genes comprise the *glv* operon (*glvA-glvR-glvC*), which is responsible for maltose dissimilation and positively regulated by maltose [29]. The significant up-regulation of these genes indicated that maltose was present in the exudates, which was confirmed by the HPLC analysis (Figure 1).

The genes involved in inositol metabolism (*iolA, iolB, iolC, iolD, iolE, iolF, iolG, iolI, iolS*) were also up-regulated, mainly with a fold change of ≥2.0 (Figure 6). Except *iolS,* which is involved in the regulation of inositol catabolism, the other eight genes are members of the *iol* operon. The increased transcription of *iolA* and *iolD* was further confirmed by real-time PCR whereas the enhancement of *iolB* and *iolL* was validated by a proteomics approach (unpublished data). The activation of nine genes indicated the presence of inositol in the exudates, which has also been verified by HPLC.

ii) A second group of genes with a higher fold change were those associated with sensing, chemotaxis, motility and biofilm formation (Table 2). These processes are crucial for bacterial colonization of roots. The recognition of signals released from roots and rhizobacteria is the first step of the establishment of a mutual cross-talk [30]. Once plant signals have been perceived, bacteria move towards the plant root to establish in the rhizosphere [31-34]. Bacterial motility in the rhizosphere involves several processes such as chemotaxis, flagella-driven motility, swarming, and production of surfactants [35-38]. The observed transcriptional changes of genes required for chemotaxis (*cheC, cheD*) and motility (*hag, fliD, fliP* and *flgM*) indicated that root exudates contain compounds that induce attraction of FZB42 cells to roots.

**Table 2:** FZB42 genes significantly induced by maize root exudates and involved in mobility and chemotaxis (Refer to experiment “Response to RE”: E-MEXP-3421)

**Gene**	**Fold change**	**Classification code_function involved**
*fliM*	2.0	1.5_ Mobility and chemotaxis
*fliP*	1.7	1.5_ Mobility and chemotaxis
*cheC*	1.7	1.5_ Mobility and chemotaxis
*cheD*	−1.5	1.5_ Mobility and chemotaxis
*hag*	3.6	1.5_ Mobility and chemotaxis
*flgM*	1.7	1.5_ Mobility and chemotaxis
*luxS*	1.7	1.3_ Sensors (signal transduction)
*ymcA*	2.5	1.3_ Sensors (signal transduction)

Biofilm formation has been documented to be involved in directing or modulating efficient colonization by PGPR [39,40]. Biofilms can also provide the plant root system with a protective barrier against attack of pathogenic microbes [35]. Two *B. amyloliquefaciens* genes involved in biofilm formation, *ycmA* and *luxS*, were enhanced by maize root exudates (Table 2, Additional file 1: Table S1). The gene *luxS*, required for synthesis of the quorum-sensing signaling molecule autoinducer-2 (AI-2) [41], is involved in biofilm formation of pathogenic *Streptococcus* species [42-44] and the probiotic *B. subtilis* natto [45]. The gene *ycmA* has also been indentified to be involved in facilitating biofilm formation [46,47]. The increased transcription of *luxS* and *ycmA* indicated that biofilm formation of FZB42 could be enhanced by some compounds present in root exudates.

iii) The third functional group with the highest number of genes induced by root exudates was associated with the non-ribosomal synthesis of secondary metabolites with antimicrobial action (Table 3). Producing secondary metabolites suppressing deleterious microbes in the rhizosphere is an established mechanism of biocontrol adopted by *B. amyloliquefaciens* FZB42 on plants [19,48,49]. The majority of the induced genes are devoted to the synthesis of two polyketide antibiotics, bacillaene and difficidin. Some components in the exudates could stimulate the production of these two antibiotics, which have been demonstrated to be able to protect orchard trees from fire blight disease caused by *Erwinia amylovora *[49].

**Table 3:** FZB42 genes which were significantly induced by maize root exudates and involved in antibiotic production (Refer to experiment “Response to RE”: E-MEXP-3421)

**Gene**	**Product**	**Fold change**
*baeE*	malonyl-CoA-[acyl-carrier protein] transacylase BaeE	1.6
*baeI*	enoyl-CoA-hydratase BaeI	2.2
*baeL*	polyketide synthase BaeL	1.9
*baeN*	hybrid NRPS/PKS BaeN	1.5
*baeR*	polyketide synthase BaeR	2.3
*dfnJ*	modular polyketide synthase of type I DfnJ	2
*dfnI*	modular polyketide synthase of type I DfnI	1.7
*dfnG*	modular polyketide synthase of type I DfnG	2
*dfnF*	modular polyketide synthase of type I DfnF	2.4
*mlnH*	polyketide synthase of type I MlnH	1.5
*fenE*	fengycin synthetase FenE	1.5
*srfAD*	surfactin synthetase D SrfAD	1.9
*srfAC*	surfactin synthetase C SrfAC	1.7

Another two genes, *mlnH* and *fenE*, were also induced, which are known to participate in non-ribosmal biosynthesis of macrolactin and fengycin, respectively. Macrolactin, a polyketide product found in FZB42, has activity against some Gram-positive bacteria [50], while fengycin can act against phytopathogenic fungi in a synergistic manner with bacillomycin D [19,51].

In addition, two genes encoding surfactin synthetase were also activated by root exudates (Table 3). Surfactin is one of *Bacillus* cyclic lipopeptides, displaying antiviral and antibacterial activities. In *Arabidopsis* it has been shown that the ability of *Bacillus* to synthesize surfactin can reduce the invasion of *Pseudomonas syringae*[30]. although it is not yet clear whether the protective effect resulted directly from the antibacterial activity of surfactin or from its biofilm-related properties. Surfactin is crucially involved in the motility of *Bacillus* by reducing surface tensions [36,37,52] and contributing to biofilm formation on *Arabidopsis* roots [30]. It has also been demonstrated that surfactin production of FZB42 was enhanced when colonizing the duckweed plant *Lemna minor *[21]. It can be expected that up-regulation of *srfAC* and *srfAD* may contribute to the protective role of surfactin against plant pathogens.

### The regulated genes with putative function

Among the 302 genes significantly altered in transcription by root exudates, 44 were annotated to encode a putative enzyme or a hypothetical protein. Similar to the genes with known function, these 44 genes fell into three categories: metabolism of carbohydrates and related molecules, metabolism of amino acids and related molecules, and transport/binding proteins and lipoproteins (Additional file 1: Table S2). Some of the 44 genes were closely associated with plant-microbe interactions. For example, the transcription of *ydjL*, nowadays renamed *bdhA*, encoding acetoin reductase/butanediol dehydrogenase [53], was 1.5-fold enhanced by root exudates. 2, 3-Butanediol is a volatile organic compound released by PGPR and able to promote significantly plant growth [54]. The expression of the gene *epsE*, residing in a 15-gene operon *epsA-O*, was also enhanced by root exudates. EpsE is involved in formation of biofilm by arresting flagellar rotation of cells embedded in biofilm matrix [55]. Another activated gene was *dfnY*, which encodes a hypothetical protein. Like other induced genes known to be involved in antibiotic production such as *dfnFdfnGdfnI* and *dfnJ* (Table 3), *dfnY* is part of the gene cluster responsible for synthesis of the polyketide antibiotic difficidin. It is worth mentioning that antibiotic production is energetically very costly and its strict control is a clear evolutionary advantage.

In contrast to a few genes significantly altered during the exponential phase (OD1.0), hundreds of genes were differentially expressed in presence of root exudates during transition to stationary growth phase (OD3.0). Such a difference may not be surprising. The transcription of most bacterial genes during the exponential growth phase is typically initiated by RNA polymerase holoenzyme carrying the housekeeping transcription factor σ^A^, while in the stationary phase, transcription is mainly accomplished by RNAP carrying alternative sigma factors allowing to adapt to a permanently changing environment. The extracytoplasmic-function (ECF) sigma factor W was enhanced in presence of root-exudate (Figure 5). SigW is known as being expressed in early stationary growth-phase and induced by various cell wall antibiotics, alkaline shock, and other stresses affecting the cell envelope. It controls a large “antibiosis” regulon involved in mediating resistance to various antibiotics including fosfomycin and the antibiotic peptides sublancin and SdpC [56]. It has been observed that many virulence-associated factors influence the colonization, persistence and spreading mechanisms of the human pathogen *Streptococcus pyogenes* in a growth phase-dependent manner [57-59]. Likewise, rhizobacteria may employ an early stationary phase-related mechanism to favor expression of those genes that mediate rhizosphere competence.

### Effect of soil extract

To simulate in part the conditions that bacteria experience in the rhizosphere soil, 10% soil extract was added to the culture media. Additional microarray experiments were performed in a similar way as before to investigate the effect of the soil extract on gene expression of FZB42. The result showed that no gene was significantly up-regulated by the soil extract during exponential growth phase of OD1.0, whereas five genes were repressed in the presence of the soil extract at OD3.0 (Table 4). This negligible number of genes that were differentially transcribed indicates that the supplement of a soil extract did not have major effects on gene transcription under the growth conditions used.

**Table 4:** FZB42 genes repressed by soil extract at OD3.0 (Refer to experiment “Response to SE”: E-MEXP-3551)

**Gene**	**Fold change**	**Product**	**Function involved**
*ypeQ*	−2.6	hypothetical protein YpeQ	unknown
*yurV*	−2.4	iron-sulfur cofactor synthesis protein nifU homolog YurV	miscellaneous
*iolS*	−2.2	inositol utilization protein S (IolS)	metabolism of carbohydrates and related molecules
*yaaA*	−2.0	conserved hypothetical protein YaaA	unknown
*ahpF*	−2.0	alkyl hydroperoxide reductase (large subunit) and NADH dehydrogenase AhpF	detoxification

### Effect of exudates prepared from maize plants colonized by FZB42

Typically, most root exudates studied were collected from plants grown in axenic systems. The release of root exudates is not only determined by the plant species, but also by plant age, physiological status, and the biotic environment that plants thrive including the rhizosphere microflora that influence the composition and quantity of root exudates [60-66]. It was reported that *P. aeruginosa* produces *N*-acyl homoserine lactone (AHL) signaling compounds that induce changes in the root exudation of *Medicago truncatula [*[67]. Exudate compounds that are specifically induced or repressed by rhizobacteria may in turn affect bacterial gene expression. Such an effect cannot be demonstrated using root exudates collected from a gnotobiotic system, therefore, a batch of “interaction exudates (IE)” was collected from maize roots which were previously inoculated with FZB42.

The transcriptional responses of FZB42 to the IE were compared with responses to the root exudates (RE) collected from axenic culture. No significant differences (q ≤ 0.01 and FCH ≥ 1.5) were found between the effect of IE and RE at OD1.0, while four genes were differentially expressed at OD3.0 (Additional file 2: Table S5). When a less stringent selection filter was applied (q ≤ 0.05 and FCH ≥ 1.5), a total of nine genes were differentially expressed (Additional file 2: Table S5). The four genes, significantly enhanced in presence of FZB42 at maize roots, encode enzymes involved in the degradation of macromolecules or cellular compounds, such as *ggt*, *nprE*, *clpP*, RBAM00438 (*ycsN*). Among all four genes, expression of the *ggt* gene was found most enhanced, bearing a fold change of 2.2 in presence of the rhizobacterium (Additional file 2: Table S5). GGT, γ-glutamyltranspeptidase (GGT) (EC 2.3.2.2) catalyzes the hydrolysis of γ-glutamyl compounds, such as glutathione (GSH), and the transfer of γ-glutamyl moieties to amino acids and peptides. The *nprE* gene, which is mainly expressed during early stationary phase, encodes extracellular neutral protease involved in degradation of proteins and peptides. The peptidase ClpP, encoded by the *clpP* gene, can associate with the ATPases ClpC, ClpE, and ClpX, thereby forming a substrate specific channel for several regulatory proteins directing spore formation or genetic competence in bacilli. RBAM00438 is a member of the aldo-keto reductases (AKRs) superfamily of soluble NAD(P)(H) oxidoreductases whose chief purpose is to reduce aldehydes and ketones to primary and secondary alcohols. At present, it remains questionable if those gene products are linked with any specific process triggered by the IE. The number of the genes obtained was much less than expected. We conclude that possible differences between the transcriptome responses to these two exudate samples are either very rare or too subtle to be revealed sufficiently by two-color microarrays.

One drawback of the present investigation is that some effects of the root exudates may have been masked by components of the 1 C medium and therefore did not result in altered gene expression. On the other hand, using 0.25 mg exudates per ml medium, some components in the exudates may have been diluted to a level at which they no longer show detectable effect on bacterial gene expression. It has been reported that the rhizosphere is a very heterogeneous soil volume, with some regions being “hotspots” of root exudation and bacterial colonization. In natural environments, bacterial populations are likely to be exposed to different concentration of exudates along the root axis [68,69].

It needs to be mentioned that the exudates used in this study were a pooled mixture of the samples collected within seven days from maize roots (see Methods). It has not yet been described to which extent the composition of root exudates is affected by the developmental stage of a plant [70] and therefore the presented bacterial responses cannot be assigned to a particular physiological state of the host plant. This question may be addressed by performing bacterial transcriptome analyses in response to exudates collected at different time points during plant development. Such an approach may be helpful to elucidate the progression of the plant-bacteria association during the plant development.

In summary, this microarray work reflects the interactions between a Gram-positive rhizobacterium and its host plant in a genome-scale perspective. Critical target genes and pathways for further investigations of the interaction were identified. Given the limited reports on transcriptomic analysis of rhizobacteria in response to their host plants [13-15], the results provided a valuable insight into PGPR behaviour in the rhizosphere. About 10% of the total number of genes were found up-regulated in presence of root exudate during transition to stationary growth phase. In addition to the findings corroborating previous transcriptome analyses performed in Gram-negative bacteria, we could demonstrate that presence of root exudate induced expression of numerous genes involved in non-ribosomal synthesis of secondary metabolites with antifungal and antibacterial action. We hypothesize that competitive colonization at plant root surfaces by FZB42 might be supported by enhanced synthesis of antimicrobial compounds.

## Conclusions

Using the data from six independent micro array experiments, differentially transcribed genes of the PGPR *B. amyloliquefaciens* FZB42 were identified and their known or putative functions were related to their associative behavior with regard to interactions with maize roots. A large group of genes specifically expressed suggested that root exudates serve primarily as a source of carbon and energy for FZB42. Another group of genes significantly induced by plant root exudates encode the non-ribosomal synthesis of antimicrobial secondary metabolites. It is possible that enhanced synthesis of antimicrobial compounds might suppress the competing phytopathogenic organisms growing within the plant rhizosphere. However, direct evidence for occurrence of those compounds in vicinity of plant rhizosphere remains to be accomplished. The addition of soil extracts to the growth medium showed no major effect on gene expression of FZB42. Similarly, the results obtained with the “interaction exudates” collected from the maize roots inoculated with FZB42 did not indicate altered effects on gene expression compared with that of common root exudates collected in the gnotobiotic system.

## Methods

### Root exudates collection and analysis

Maize seeds (Saaten-Union, Germany) were surface-sterilized and germinated as described previously [21]. Root exudates were collected from the maize seedlings grown in an axenic system with sterile water (1:1 distilled water and tap water, v/v). Forty germinated seeds harboring a main root of at least 2 cm length were transferred into test tubes filled with 2 ml of autoclaved water, with the maize seeds being placed just above the water surface. The tubes were kept under sterile conditions and maintained in a plant growth room (16-h light/8-h dark) at 24°C for 8 days. In the first two days, water was supplemented to the tubes, and seedlings were pulled to a higher position to ensure that the maize seeds were always above the water surface as the roots elongated. From the third day on, the water containing the exudates was collected and the tubes were refilled with sterile water. Sampling was performed every day until the eighth day after transferring the seedlings. Each collection were kept separate, from which a 100 μL aliquot was taken and spread on a solid LB media to check for contamination. The contaminated samples were discarded.

To collect the “interaction exudates (IE)”, the germinated maize seeds were inoculated with FZB42 as described previously [21] before transferring the test tubes. Afterward the maize was grown and the exudates were prepared in the same way as described above.

The collected exudates were pooled, freeze-dried and stored at −20°C. Before use, the lyophilized exudates were weighted, and dissolved in a certain volume of distilled water. The obtained exudates solution was centrifuged to remove any insoluble constituents. The supernatant was filter-sterilized and the resulting stock exudates were stored in dark at −80°C. The final concentration of the exudates in the culture vessel was generally adjusted to 0.25 g L^-1^. Chemical analysis of the root exudates was performed as described previously [71]: amino acids were determined using a Shimadzu HPLC system. 40 μL samples were derivatized with 160 μl OPA (o-phthaldialdehyde) reagent and 20 μL of the resulting mixture were injected and separated on a GROM-SIL OPA-3 column using solvent gradient elution by solvent A (25 mM phosphate buffer pH 7.2 with 0.75% tetrahydrofuran) and solvent B (methanol to acetonitrile to 25 mM phosphate buffer 7.2 [35 : 15 : 50/v : v : v]). Gradient profile: 0–2 min, 0% B; 2–10 min, 0%-50% B; 10–15 min, 50–60% B; 15–20 min, 60–100% B; 20–25 min, 100% B; 25–26 min, 100%-0% B; 26–35 min, 0% B. The flow-rate was 1 mL min^-1^. Subsequent fluorescence detection of the derivatives was performed at an excitation wavelength of 330 nm and 450 nm. Organic acids were determined by means of ion chromatography (Dionex IonPac AS 11 HC column) using a gradient ranging from 4 mM to 80 mM KOH. Organic acids were identified by comparison of retention time with known standards. Sugars were determined by GC-TOF-MS. A lyophilized 75 μL aliquot of root exudates was dissolved in 50 mL methoxyamine hydrochloride in dry pyrididine and derivatized for 2 h at 37°C followed by 30 min. treatment with 50 μL N-methyl-N-trifluoroacetamide at 37°C. A volume of 1 μL was injected into the GC column.

### Microarray design

The Bam4kOLI microarray was designed based on the sequenced complete genome of *B. amyloliquefaciens* FZB42 [27] (Additional file 3: Table S6). The array contained 3931 50-70mer oligonucleotides representing predicted protein-encoding genes and a set of small non-coding RNA genes of FZB42. In addition, the array included stringency controls with 71%, 80% and 89% identity to the native sequences of five genes, *dnaArpsLrpsOrpsP*, and *rpmI*, to monitor the extent of cross hybridization. The array also contained alien DNA oligonucleotides for four antibiotic resistance genes (*Em*^*r*^*Cm*^*r*^*Nm*^*r*^ and *Spc*^*r*^) and eight spiking controls as well as one empty control (nothing spotted). All oligonucleotide probes were printed in four replicates. Microarrays were produced and processed as described previously [72].

Oligonucleotides were designed using the Oligo Designer software (Bioinformatics Resource Facility, CeBiTec, Bielefeld University). Melting temperatures of the oligonucleotides were calculated based on %GC and oligo length, ranging from 73°C to 83°C (optimal 78°C). Salt concentration was set to 0.1 M. QGramMatch was used to analyse uniqueness of the oligos.

### Experimental design

The experiment designs of FZB42 in response to various conditions are summarized in Additional file 3: Table S6. Independent experiments were used as biological replicates. In all comparisons dye-swap were carried out to minimize the effect of dye biases.

1 C medium (0.7% w/v pancreatic digest of casein, 0.3% w/v papain digest of soya flour, 0.5% w/v NaCl) containing 0.1% glucose was used in all experiments. Except the controls of the experiment “Response to SE” (Additional file 3: Table S6), 10% soil extract was also supplemented in the media. Soil extract was prepared by extracting 500 g dried, fertile garden soil with one litre distilled water for 2 hrs and autoclaving. After cooling down, the supernatant was filtered with 0.22 μm Nuclepore unit and then stored at 4°C until use.

### Total RNA preparation

One overnight colony of FZB42 was inoculated into 1 C medium plus 0.1% glucose and then shaken at 210 rpm at 24°C. After 14 hours the obtained preculture was used to inoculate a new 1 C medium (containing 0.1% glucose) plus the corresponding solution to be studied (maize root exudates, soil extract, or interaction exudates. See Additional file 3: Table S6). The main cultures were grown at 24°C until they reached late exponential growth phase (OD 1.0) and/or the transition to stationary phase (OD3.0, see Additional file 4: Figure S1).

The FZB42 cells of OD1.0 or OD3.0 were harvested for preparation of total RNA. A volume of 15 ml of the culture was mixed with 7.5 ml “killing buffer” (20 mM Tris–HCl, 5 mM MgCl2, 20 mM NaN3, pH 7.5) and then centrifuged at 5,000 rpm for 3 minutes at room temperature. The pellet was washed once more with 1 ml “killing buffer” and then immediately frozen in liquid nitrogen. The frozen cell pellets were stored at −80°C until RNA isolation.

Isolation of RNA was performed using the Nucleo Spin® RNA L (Macherey Nagel) according to the manufacturer’s instructions. The isolated RNA was additionally digested with DNaseI to avoid possible trace DNA contamination. After ethanol precipitation RNA pellets were resuspended in 300 μl RNase-free water. The concentration of total RNA was spectrophotometrically determined, whereas its quality was checked on a 1.5% RNA agarose gel in 1 × MEN buffer (20 mM MOPS; 1 mM EDTA, 5 mM NaAc; pH7.0) with 16% formaldehyde.

### Synthesis of labeled cDNA, hybridization and image acquisition

Synthesis of first-strand cDNA, microarray hybridization and image acquisition were performed in CeBiTec, the Center for Biotechnology at Bielefeld University. Briefly, aminoallyl-modified first-strand cDNAs were synthesized by reverse transcription according to DeRisi et al [73]. and then coupled with Cy3- and Cy5-*N-*hydroxysuccinimidyl ester dyes (GE Healthcare, Little Chalfont, UK). After hybridization using the HS4800 hybridization station (Tecan Trading AG, Switzerland), slides were scanned with a pixel size of 10 μm using the LS Reloaded microarray scanner (Tecan Trading AG, Switzerland).

### Data processing

The microarray data obtained was analysed by using the EMMA 2.8.2 software [74]. The mean signal intensity (*A*_i_) was calculated for each spot using the formula *A*_i_ = log_2_(*R*_i_*G*_i_)^0.5^[75]. *R*_i_ = *I*_ch1(i)_ − *Bg*_ch1(i)_ and *G*_i_ = *I*_ch2(i)_ − *Bg*_ch2(i)_, where *I*_ch1(i)_ or *I*_ch2(i)_ is the intensity of a spot in channel 1 or channel 2, and *Bg*_ch1(i)_ or *Bg*_ch2(i)_ is the background intensity of a spot in channel1 or channel 2, respectively. The log_2_ value of the ratio of signal intensities (*M*i) was calculated for each spot using the formula *M*i = log_2_(*R*i/*G*i). Spots were flagged as “empty” if *R* ≤ 0.5 in both channels, where *R* = (signal mean–background mean)/background standard deviation [76]. The raw data were normalized by the method of LOWESS (locally weighted scattered plot smoothing). A significance test was performed by the method of false discovery rate (FDR) control and the adjusted p-value defined by FDR was called q-value [77,78].

An arbitrary cutoff, fold change (FCH) greater than 1.5, was applied to the genes with a q-value of ≤0.01. Only those genes which meet both filter conditions (q ≤ 0.01 & FCH ≥ 1.5) were regarded to be significantly differentially expressed.

### Real-time PCR

The first-strand cDNA was obtained by reverse transcription with RevertAid^TM^ Premium Reverse Transcriptase (Fermentas, St. Leon-Rot, Germany), using random hexamers as primers. Oligonucleotide primers were designed by the software PrimerExpress and listed in supplemental materials (Additional files 1: Table S4). Real-time PCR was performed with SYBR® Green PCR Master Mix kit (Carlsbad, California, USA) using 7500 Fast Real-Time PCR System (Carlsbad, California, USA) according to the manufacturers’ instructions. As an internal control, the housekeeping gene *gyrA* was used as its expression was not significantly altered in all microarray experiments. Three technical replicates were carried out for each target gene. Quantification was analysed based on the threshold cycle (Ct) values as described by Pfaffl [79].

The raw data of the Micro-array experiments, described here, are available in the ArrayExpress database under the accession numbers: E-MEXP-3421, E-MEXP-3550, E-MEXP-3551, E-MEXP-3553, E-MEXP-3554, respectively (see also Additional file 3: Table S6).

## Authors’ contributions

BF carried out the main experiments and data analysis and wrote the manuscript draft. LCC performed complementary experiments and revised the manuscript. AB designed the array and was responsible for the hybridization experiments. DF performed the metabolite analysis of root exudates. NvW revised the manuscript. RB guided experimental design and wrote the final version of the manuscript. All authors read and approved the final manuscript.

## Supplementary Material

Additional file 1 **Table S1. The genes of FZB42 with known function whose transcriptions were significantly altered in response to maize root exudates at OD3.0 (Refer to experiment “Response to RE”: E-MEXP-3421).** Table S2: The genes of FZB42 with putative function or encoding hypothetical protein whose transcriptions were significantly altered in response to maize root exudates at OD3.0 (Refer to experiment “Response to RE”: E-MEXP-3421). Table S3: The genes of FZB42 with unknown function whose transcriptions were significantly altered in response to maize root exudates at OD3.0 (Refer to experiment “Response to RE”: E-MEXP-3421). Table S4: The primers used for real-time PCR. (DOCX 52 kb) Click here for file

Additional file 2 **Table S5. Differentially expressed genes of FZB42 in response to IE compared with those to RE (Refer to experiment “IE <> RE”: E-MEXP-3553).** The genes highlighted were those with a q value of ≤0.01. (DOC 33 kb) Click here for file

Additional file 3 **Table S6. Microarray experimental design and data bank accession.** (DOC 40 kb) Click here for file

Additional file 4 **Figure S2. Growth of FZB42 at 24°C under continuous shaking (220 rpm/min.) in medium 1 C supplemented with sterilized 10% soil extract prepared by extracting of 500 g (dry weight) compost soil with 1 L distilled water.** Cells were sampled during exponential growth (OD_600_ = 1.0) and during transition to stationary growth phase. The time of sampling in the transition phase (O.D._600_ = 3.0) is indicated by the red arrow. (DOC 32 kb) Click here for file

## References

[B1] Lugtenberg BJJ Kamilova F Plant-growth-promoting rhizobacteria Annu Rev Microbiol 2009 63 541 556 10.1146/annurev.micro.62.081307.162918 19575558

[B2] Kloepper JW Schroth MN Plant growth-promoting rhizobacteria on radishes Proc of the 4th Internat Conf on Plant Pathogenic Bacter 1978 INRA, Angers, France

[B3] Domenech J Reddy MS Kloepper JW Ramos B Gutierrez-Manero J Combined application of the biological product LS213 with Bacillus, Pseudomonas or Chryseobacterium for growth promotion and biological control of soil-borne diseases in pepper and tomato BioControl 2006 51 2 245 258 10.1007/s10526-005-2940-z

[B4] Alabouvette C Olivain C Migheli Q Steinberg C Microbiological control of soil-borne phytopathogenic fungi with special emphasis on wilt-inducing Fusarium oxysporum New Phytol 2009 184 3 529 544 10.1111/j.1469-8137.2009.03014.x 19761494

[B5] Dessaux Y Ryan PR Thomashow LS Weller DM Rhizosphere engineering and management for sustainable agriculture Plant Soil 2009 321 1–2 363 383

[B6] Somers E Vanderleyden J Srinivasan M Rhizosphere bacterial signalling: a love parade beneath our feet Crit Rev Microbiol 2004 30 4 205 240 10.1080/10408410490468786 15646398

[B7] Oger P Petit A Dessaux Y Genetically engineered plants producing opines alter their biological environment Nat Biotech 1997 15 4 369 372 10.1038/nbt0497-369 9094140

[B8] Rudrappa T Czymmek KJ Pare PW Bais HP Root-secreted malic acid recruits beneficial soil bacteria Plant Physiol 2008 148 3 1547 1556 10.1104/pp.108.127613 18820082 PMC2577262

[B9] Micallef SA Shiaris MP Colon-Carmona A Influence of Arabidopsis thaliana accessions on rhizobacterial communities and natural variation in root exudates J Exp Bot 2009 60 6 1729 1742 10.1093/jxb/erp053 19342429 PMC2671628

[B10] Badri DV Vivanco JM Regulation and function of root exudates Plant Cell Environ 2009 32 6 666 681 10.1111/j.1365-3040.2009.01926.x 19143988

[B11] Shi S Richardson AE O'Callaghan M DeAngelis KM Jones EE Stewart A Firestone MK Condron LM Effects of selected root exudate components on soil bacterial communities FEMS Microbiol Ecol 2011 77 3 600 610 10.1111/j.1574-6941.2011.01150.x 21658090

[B12] Diehn M Relman DA Comparing functional genomic datasets: lessons from DNA microarray analyses of host-pathogen interactions Curr Opin Microbiol 2001 4 1 95 101 10.1016/S1369-5274(00)00171-5 11173041

[B13] Mark GL Dow JM Kiely PD Higgins H Haynes J Baysse C Abbas A Foley T Franks A Morrissey J Transcriptome profiling of bacterial responses to root exudates identifies genes involved in microbe-plant interactions Proc Natl Acad Sci U S A 2005 102 48 17454 17459 10.1073/pnas.0506407102 16301542 PMC1297666

[B14] Matilla M Espinosa-Urgel M Rodriguez-Herva J Ramos J Ramos-Gonzalez M Genomic analysis reveals the major driving forces of bacterial life in the rhizosphere Genome Biol 2007 8 9 R179 10.1186/gb-2007-8-9-r179 17784941 PMC2375017

[B15] Ramachandran VK East AK Karunakaran R Downie JA Poole PS Adaptation of Rhizobium leguminosarum to pea, alfalfa and sugar beet rhizospheres investigated by comparative transcriptomics Genome Biol 2011 12 10 R106 10.1186/gb-2011-12-10-r106 22018401 PMC3333776

[B16] Bashan Y Holguin G de-Bashan LE Azospirillum-plant relationships: physiological, molecular, agricultural, and environmental advances (1997–2003) Can J Microbiol 2004 50 8 521 577 10.1139/w04-035 15467782

[B17] Steenhoudt O Vanderleyden J Azospirillum, a free-living nitrogen-fixing bacterium closely associated with grasses: genetic, biochemical and ecological aspects FEMS Microbiol Rev 2000 24 4 487 506 10.1111/j.1574-6976.2000.tb00552.x 10978548

[B18] Elizabeth ABE Jo H Biocontrol of plant disease: a (Gram-) positive perspective FEMS Microbiol Lett 1999 171 1 1 9 10.1111/j.1574-6968.1999.tb13405.x 9987836

[B19] Chen XH Koumoutsi A Scholz R Borriss R More than anticipated - production of antibiotics and other secondary metabolites by Bacillus amyloliquefaciens FZB42 J Mol Microbiol Biotechnol 2009 16 1–2 14 24 18957859 10.1159/000142891

[B20] Idris EE Iglesias DJ Talon M Borriss R Tryptophan-dependent production of indole-3-acetic acid (IAA) affects level of plant growth promotion by Bacillus amyloliquefaciens FZB42 Mol Plant Microbe Interact 2007 20 6 619 626 10.1094/MPMI-20-6-0619 17555270

[B21] Fan B Chen XH Budiharjo A Bleiss W Vater J Borriss R Efficient colonization of plant roots by the plant growth promoting bacterium Bacillus amyloliquefaciens FZB42, engineered to express green fluorescent protein J Biotechnol 2011 151 4 303 311 10.1016/j.jbiotec.2010.12.022 21237217

[B22] Lugtenberg BJJ Dekkers LC Bloemberg GV Molecular determinants of rhizosphere colonization by Pseudomonas Annu Rev Phytopathol 2001 39 461 490 10.1146/annurev.phyto.39.1.461 11701873

[B23] Lugtenberg BJJ Dekkers LC What makes Pseudomonas bacteria rhizosphere competent? Environ Microbiol 1999 1 1 9 13 10.1046/j.1462-2920.1999.00005.x 11207713

[B24] Simons M van der Bij AJ Brand I de Weger LA Wijffelman CA Lugtenberg BJ Gnotobiotic system for studying rhizosphere colonization by plant growth-promoting Pseudomonas bacteria Mol Plant Microbe Interact 1996 9 7 600 607 10.1094/MPMI-9-0600 8810075

[B25] Kraffczyk I Trolldenier G Beringer H Soluble root exudates of maize: Influence of potassium supply and rhizosphere microorganisms Soil Biol Biochem 1984 16 4 315 322 10.1016/0038-0717(84)90025-7

[B26] Dennis PG Miller AJ Hirsch PR Are root exudates more important than other sources of rhizodeposits in structuring rhizosphere bacterial communities? FEMS Microbiol Ecol 2010 72 3 313 327 10.1111/j.1574-6941.2010.00860.x 20370828

[B27] Chen XH Koumoutsi A Scholz R Eisenreich A Schneider K Heinemeyer I Morgenstern B Voss B Hess WR Reva O Comparative analysis of the complete genome sequence of the plant growth-promoting bacterium Bacillus amyloliquefaciens FZB42 Nat Biotechnol 2007 25 9 1007 1014 10.1038/nbt1325 17704766

[B28] Moszer I Jones LM Moreira S Fabry C Danchin A SubtiList: the reference database for the Bacillus subtilis genome Nucleic Acids Res 2002 30 1 62 65 10.1093/nar/30.1.62 11752255 PMC99059

[B29] Yamamoto H Serizawa M Thompson J Sekiguchi J Regulation of the glv operon in Bacillus subtilis: YfiA (GlvR) is a positive regulator of the operon that is repressed through CcpA and cre J Bacteriol 2001 183 17 5110 5121 10.1128/JB.183.17.5110-5121.2001 11489864 PMC95387

[B30] Bais HP Fall R Vivanco JM Biocontrol of Bacillus subtilis against infection of Arabidopsis roots by Pseudomonas syringae is facilitated by biofilm formation and surfactin production Plant Physiol 2004 134 1 307 319 10.1104/pp.103.028712 14684838 PMC316310

[B31] de Weert S Vermeiren H Mulders IH Kuiper I Hendrickx N Bloemberg GV Vanderleyden J De Mot R Lugtenberg BJ Flagella-driven chemotaxis towards exudate components is an important trait for tomato root colonization by Pseudomonas fluorescens Mol Plant Microbe Interact 2002 15 11 1173 1180 10.1094/MPMI.2002.15.11.1173 12423023

[B32] De Weert S Kuiper I Lagendijk EL Lamers GE Lugtenberg BJ Role of chemotaxis toward fusaric acid in colonization of hyphae of Fusarium oxysporum f. sp. radicis-lycopersici by Pseudomonas fluorescens WCS365 Mol Plant Microbe Interact 2004 17 11 1185 1191 10.1094/MPMI.2004.17.11.1185 15553244

[B33] O'Sullivan DJ O'Gara F Traits of fluorescent Pseudomonas spp. involved in suppression of plant root pathogens Microbiol Rev 1992 56 4 662 676 1480114 10.1128/mr.56.4.662-676.1992 PMC372893

[B34] Walsh UF Morrissey JP O'Gara F Pseudomonas for biocontrol of phytopathogens: from functional genomics to commercial exploitation Curr Opin Biotechnol 2001 12 3 289 295 10.1016/S0958-1669(00)00212-3 11404107

[B35] Ongena M Jacques P Bacillus lipopeptides: versatile weapons for plant disease biocontrol Trends Microbiol 2008 16 3 115 125 10.1016/j.tim.2007.12.009 18289856

[B36] Raaijmakers JM de Bruijn I de Kock MJ Cyclic lipopeptide production by plant-associated Pseudomonas spp.: diversity, activity, biosynthesis, and regulation Mol Plant Microbe Interact 2006 19 7 699 710 10.1094/MPMI-19-0699 16838783

[B37] Daniels R Vanderleyden J Michiels J Quorum sensing and swarming migration in bacteria FEMS Microbiol Rev 2004 28 3 261 289 10.1016/j.femsre.2003.09.004 15449604

[B38] Capdevila S Martinez-Granero FM Sanchez-Contreras M Rivilla R Martin M Analysis of Pseudomonas fluorescens F113 genes implicated in flagellar filament synthesis and their role in competitive root colonization Microbiology 2004 150 Pt 11 3889 3897 15528673 10.1099/mic.0.27362-0

[B39] Combes-Meynet E Pothier JF Moenne-Loccoz Y Prigent-Combaret C The Pseudomonas secondary metabolite 2,4-diacetylphloroglucinol is a signal inducing rhizoplane expression of Azospirillum genes involved in plant-growth promotion Mol Plant Microbe Interact 2010 24 2 271 284 21043573 10.1094/MPMI-07-10-0148

[B40] Ramey BE Koutsoudis M Bodman SBv Fuqua C Biofilm formation in plant-microbe associations Curr Opin Microbiol 2004 7 6 602 609 10.1016/j.mib.2004.10.014 15556032

[B41] Surette MG Miller MB Bassler BL Quorum sensing in Escherichia coli, Salmonella typhimurium, and Vibrio harveyi: a new family of genes responsible for autoinducer production Proc Natl Acad Sci U S A 1999 96 4 1639 1644 10.1073/pnas.96.4.1639 9990077 PMC15544

[B42] Heilmann C Schweitzer O Gerke C Vanittanakom N Mack D Gotz F Molecular basis of intercellular adhesion in the biofilm-forming Staphylococcus epidermidis Mol Microbiol 1996 20 5 1083 1091 10.1111/j.1365-2958.1996.tb02548.x 8809760

[B43] Gotz F Staphylococcus and biofilms Mol Microbiol 2002 43 6 1367 1378 10.1046/j.1365-2958.2002.02827.x 11952892

[B44] Huang Z Meric G Liu Z Ma R Tang Z Lejeune P luxS-based quorum-sensing signaling affects Biofilm formation in Streptococcus mutans J Mol Microbiol Biotechnol 2009 17 1 12 19 10.1159/000159193 18818488

[B45] Lombardia E Rovetto AJ Arabolaza AL Grau RR A LuxS-dependent cell-to-cell language regulates social behavior and development in Bacillus subtilis J Bacteriol 2006 188 12 4442 4452 10.1128/JB.00165-06 16740951 PMC1482974

[B46] Branda SS Gonzalez-Pastor JE Dervyn E Ehrlich SD Losick R Kolter R Genes involved in formation of structured multicellular communities by Bacillus subtilis J Bacteriol 2004 186 12 3970 3979 10.1128/JB.186.12.3970-3979.2004 15175311 PMC419949

[B47] Kearns DB Chu F Branda SS Kolter R Losick R A master regulator for biofilm formation by Bacillus subtilis Mol Microbiol 2005 55 3 739 749 15661000 10.1111/j.1365-2958.2004.04440.x

[B48] Chen XH Koumoutsi A Scholz R Schneider K Vater J Sussmuth R Piel J Borriss R Genome analysis of Bacillus amyloliquefaciens FZB42 reveals its potential for biocontrol of plant pathogens J Biotechnol 2009 140 1–2 27 37 19041913 10.1016/j.jbiotec.2008.10.011

[B49] Chen XH Scholz R Borriss M Junge H Mogel G Kunz S Borriss R Difficidin and bacilysin produced by plant-associated Bacillus amyloliquefaciens are efficient in controlling fire blight disease J Biotechnol 2009 140 1–2 38 44 19061923 10.1016/j.jbiotec.2008.10.015

[B50] Schneider K Chen XH Vater J Franke P Nicholson G Borriss R Sussmuth RD Macrolactin is the polyketide biosynthesis product of the pks2 cluster of Bacillus amyloliquefaciens FZB42 J Nat Prod 2007 70 9 1417 1423 10.1021/np070070k 17844999

[B51] Koumoutsi A Chen XH Henne A Liesegang H Hitzeroth G Franke P Vater J Borriss R Structural and functional characterization of gene clusters directing nonribosomal synthesis of bioactive cyclic lipopeptides in Bacillus amyloliquefaciens strain FZB42 J Bacteriol 2004 186 4 1084 1096 10.1128/JB.186.4.1084-1096.2004 14762003 PMC344220

[B52] Leclere V Marti R Bechet M Fickers P Jacques P The lipopeptides mycosubtilin and surfactin enhance spreading of Bacillus subtilis strains by their surface-active properties Arch Microbiol 2006 186 6 475 483 10.1007/s00203-006-0163-z 16964493

[B53] Nicholson WL The Bacillus subtilis ydjL (bdhA) gene encodes acetoin reductase/2,3-butanediol dehydrogenase Appl Environ Microbiol 2008 74 22 6832 6838 10.1128/AEM.00881-08 18820069 PMC2583490

[B54] Ryu CM Farag MA Hu CH Reddy MS Wei HX Pare PW Kloepper JW Bacterial volatiles promote growth in Arabidopsis Proc Natl Acad Sci U S A 2003 100 8 4927 4932 10.1073/pnas.0730845100 12684534 PMC153657

[B55] Blair KM Turner L Winkelman JT Berg HC Kearns DB A molecular clutch disables flagella in the Bacillus subtilis biofilm Science 2008 320 5883 1636 1638 10.1126/science.1157877 18566286

[B56] Mascher T Hachmann AB Helmann JD Regulatory overlap and functional redundancy among Bacillus subtilis extracytoplasmic function sigma factors J Bacteriol 2007 189 19 6919 6927 10.1128/JB.00904-07 17675383 PMC2045236

[B57] Kreikemeyer B McIver KS Podbielski A Virulence factor regulation and regulatory networks in Streptococcus pyogenes and their impact on pathogen-host interactions Trends Microbiol 2003 11 5 224 232 10.1016/S0966-842X(03)00098-2 12781526

[B58] Beyer-Sehlmeyer G Kreikemeyer B Horster A Podbielski A Analysis of the growth phase-associated transcriptome of Streptococcus pyogenes Int J Med Microbiol 2005 295 3 161 177 10.1016/j.ijmm.2005.02.010 16044856

[B59] Chaussee MA Dmitriev AV Callegari EA Chaussee MS Growth phase-associated changes in the transcriptome and proteome of Streptococcus pyogenes Arch Microbiol 2008 189 1 27 41 17665172 10.1007/s00203-007-0290-1

[B60] Wieland G Neumann R Backhaus H Variation of microbial communities in soil, rhizosphere, and rhizoplane in response to crop species, soil type, and crop development Appl Environ Microbiol 2001 67 12 5849 5854 10.1128/AEM.67.12.5849-5854.2001 11722945 PMC93382

[B61] Buyer JS Roberts DP Russek-Cohen E Soil and plant effects on microbial community structure Can J Microbiol 2002 48 11 955 964 10.1139/w02-095 12556123

[B62] Kowalchuk GA Buma DS de Boer W Klinkhamer PG van Veen JA Effects of above-ground plant species composition and diversity on the diversity of soil-borne microorganisms Antonie van Leeuwenhoek 2002 81 1–4 509 520 12448746 10.1023/a:1020565523615

[B63] Broeckling CD Broz AK Bergelson J Manter DK Vivanco JM Root exudates regulate soil fungal community composition and diversity Appl Environ Microbiol 2008 74 3 738 744 10.1128/AEM.02188-07 18083870 PMC2227741

[B64] Kuzyakov Y Raskatov A Kaupenjohann M Turnover and distribution of root exudates of Zea mays Plant Soil 2003 254 2 317 327 10.1023/A:1025515708093

[B65] Yang CH Crowley DE Rhizosphere microbial community structure in relation to root location and plant iron nutritional status Appl Environ Microbiol 2000 66 1 345 351 10.1128/AEM.66.1.345-351.2000 10618246 PMC91828

[B66] Wang Y Ohara Y Nakayashiki H Tosa Y Mayama S Microarray analysis of the gene expression profile induced by the endophytic plant growth-promoting rhizobacteria, Pseudomonas fluorescens FPT9601-T5 in Arabidopsis Mol Plant Microbe Interact 2005 18 5 385 396 10.1094/MPMI-18-0385 15915637

[B67] Mathesius U Mulders S Gao M Teplitski M Caetano-Anolles G Rolfe BG Bauer WD Extensive and specific responses of a eukaryote to bacterial quorum-sensing signals Proc Natl Acad Sci U S A 2003 100 3 1444 1449 10.1073/pnas.262672599 12511600 PMC298792

[B68] Dennis PG Miller AJ Hirsch PR Are root exudates more important than other sources of rhizodeposits in structuring rhizosphere bacterial communities? FEMS Microbiol Ecol 72 3 313 327 20370828 10.1111/j.1574-6941.2010.00860.x

[B69] Kuzyakov Y Priming effects: Interactions between living and dead organic matter Soil Biol Biochem 2010 42 9 1363 1371 10.1016/j.soilbio.2010.04.003

[B70] Haichar FZ Marol C Berge O Rangel-Castro JI Prosser JI Balesdent J Heulin T Achouak W Plant host habitat and root exudates shape soil bacterial community structure ISME J 2008 2 12 1221 1230 10.1038/ismej.2008.80 18754043

[B71] Carvalhais LC Dennis PG Fedoseyenko D Hajirezaei MR Borriss R von Wiren N Root exudation of sugars, amino acids, and organic acids by maize as affected by nitrogen, phosphorus, potassium, and iron deficiency Journal of Plant Nutrition and Soil Science 2011 174 1 3 11 10.1002/jpln.201000085

[B72] Brune I Becker A Paarmann D Albersmeier A Kalinowski J Puhler A Tauch A Under the influence of the active deodorant ingredient 4-hydroxy-3-methoxybenzyl alcohol, the skin bacterium Corynebacterium jeikeium moderately responds with differential gene expression J Biotechnol 2006 127 1 21 33 10.1016/j.jbiotec.2006.06.011 16890319

[B73] DeRisi JL Iyer VR Brown PO Exploring the metabolic and genetic control of gene expression on a genomic scale Science 1997 278 5338 680 686 10.1126/science.278.5338.680 9381177

[B74] Dondrup M Albaum SP Griebel T Henckel K Junemann S Kahlke T Kleindt CK Kuster H Linke B Mertens D EMMA 2–a MAGE-compliant system for the collaborative analysis and integration of microarray data BMC Bioinforma 2009 10 50 10.1186/1471-2105-10-50 PMC2645365 19200358

[B75] Dudoit S Yang YH Callow MJ Speed TP Statistical methods for identifying differentially expressed genes in replicated cDNA microarray experiments Stat Sin 2002 12 1 111 139

[B76] Serrania J Vorholter FJ Niehaus K Puhler A Becker A Identification of Xanthomonas campestris pv. campestris galactose utilization genes from transcriptome data J Biotechnol 2008 135 3 309 317 10.1016/j.jbiotec.2008.04.011 18538881

[B77] Benjamini Y Hochberg Y Controlling the False Discovery Rate: A Practical and Powerful Approach to Multiple Testing Journal of the Royal Statistical Society Series B (Methodological) 1995 57 1 289 300

[B78] Roberts PC El-Gewely MR Gene expression microarray data analysis demystified Biotechnol Annu Rev 2008 14 29 61 18606359 10.1016/S1387-2656(08)00002-1

[B79] Pfaffl MW A new mathematical model for relative quantification in real-time RT-PCR Nucleic Acids Res 2001 29 9 e45 10.1093/nar/29.9.e45 11328886 PMC55695

