# Supplementary material for: Transcriptomic profiling of Bacillus amyloliquefaciens FZB42 in response to maize root exudates
Source: BMC Microbiol. 2012 Jun 21;12:116. doi: 10.1186/1471-2180-12-116 (PMC3438084; doi:10.1186/1471-2180-12-116)
Supplement: Additional file 1 — Table S1. The genes of FZB42 with known function whose transcriptions were significantly altered in response to maize root exudates at OD3.0 (Refer to experiment “Response to RE”: E-MEXP-3421). Table S2: The genes of FZB42 with putative function or encoding hypothetical protein whose transcriptions were significantly altered in response to maize root exudates at OD3.0 (Refer to experiment “Response to RE”: E-MEXP-3421). Table S3: The genes of FZB42 with unknown function whose transcriptions were significantly altered in response to maize root exudates at OD3.0 (Refer to experiment “Response to RE”: E-MEXP-3421). Table S4: The primers used for real-time PCR. (DOCX 52 kb) [file 1471-2180-12-116-S1.docx]

### SM Table 1: The genes of FZB42 with known function whose transcriptions were significantly altered in response to maize root exudates at OD3.0 (Refer to experiment “Response to RE”: E-MEXP-3421).

| ***Gene*** | ***FCH*** |  |  | ***Product*** | |
| --- | --- | --- | --- | --- | --- |
|  |  |  |  |  | |
|  |  | **1_Cell envelope and cellular processes** | | | |
|  |  |  | **1.7_ Cell division** | | |
| *divIC* | 1.7 |  |  | cell-division initiation protein DivIC | |
| *ftsH* | 1.5 |  |  | cell division protein and general stress protein(class III heat-shock protein) FtsH | |
| *ftsL* | 1.7 |  |  | cell-division protein FtsL | |
| *ftsZ* | 1.7 |  |  | cell-division initiation protein FtsZ | |
| *minC* | 1.6 |  |  | cell-division inhibitor (septum placement) MinC | |
| *ywkC* | -1.5 |  |  | cell division protein: attaches the chromosome to the cell pole | |
|  |  |  | **1.1_ Cell wall** | | |
| *pbpF* | 1.5 |  |  | penicillin-binding protein 2C PbpF | |
| *murB* | 1.6 |  |  | UDP-N-acetylenolpyruvoylglucosamine reductase MurB | |
| *tuaB* | -1.5 |  |  | teichuronic acid biosynthesis protein TuaB | |
| *ymfM* | 1.5 |  |  | required for cell shape determination | |
| *yoeB* | 1.6 |  |  | inhibits in vitro activity of cell wall endopeptidases LytE and LytF, inhibits cell separation | |
|  |  |  | **1.4_ Membrane bioenergetics** | | |
| *yjlD* | 1.6 |  |  | NADH dehydrogenase-like protein YjlD | |
| *resA* | 1.7 |  |  | thiol-disulfide oxidoreductase ResA | |
| *atpC* | 1.6 |  |  | ATP synthase (subunit epsilon) AtpC | |
| *atpH* | 1.7 |  |  | ATP synthase (subunit delta) AtpH | |
| *atpF* | 1.5 |  |  | ATP synthase (subunit B) AtpF | |
| *qoxB* | 1.6 |  |  | quinol oxidase polypeptide I QoxB | |
| *qoxA* | 1.6 |  |  | quinol oxidase subunit II precursor QoxA | |
|  |  |  | **1.5_ Mobility and chemotaxis** | | |
| *fliM* | 2.0 |  |  | flagellar motor switch protein FliM | |
| *fliP* | 1.7 |  |  | flagellar biosynthetic protein FliP | |
| *cheC* | 1.7 |  |  | chemotaxis protein CheC | |
| *cheD* | -1.5 |  |  | chemotaxis protein CheD | |
| *hag* | 3.6 |  |  | flagellin proteinHag | |
| *flgM* | 1.7 |  |  | negative regulator of flagellin synthesis (Anti-sigma-D factor) FlgM | |
|  |  |  | **1.3_ Sensors (signal transduction)** | | |
| *luxS* | 1.7 |  |  | s-ribosylhomocysteine lyase LuxS | |
| *ymcA* | 2.5 |  |  | antagonist of biofilm repression by SinR, regulation of biofilm formation | |
|  |  |  | **1.6_ Protein secretion** | | |
| *secE* | 1.7 |  |  | preprotein translocase subunit SecE | |
| *secY* | 2.0 |  |  | preprotein translocase subunit SecY | |
| *tatAy* | 1.6 |  |  | sec-independent protein translocase protein TatAy | |
| *tatCy* | 1.6 |  |  | sec-independent protein translocase protein TatCy | |
| *lytA* | 1.5 |  |  | membrane bound lipoprotein LytA | |
|  |  |  | **1.8_ Sporulation** | | |
| *rapA* | 1.7 |  |  | response regulator aspartate phosphatase RapA | |
| *ypeB* | 1.5 |  |  | sporulation protein YpeB | |
| *sda* | 1.7 |  |  | sporulation inhibitor Sda | |
| *spoIIB* | -1.7 |  |  | endospore development protein SpoIIB | |
| *sspI* | -1.7 |  |  | small acid-soluble spore protein SspI | |
| *cotG* | 1.7 |  |  | spore coat protein G (CotG) | |
| *yabP* | 2.1 |  |  | required for sporulation at a late stage | |
|  |  |  | **1.1_ Transformation/competence** | | |
| *comS* | 1.7 |  |  | competence protein S ComS | |
| *med* | -1.6 |  |  | transcriptional activator protein med precursor Med | |
|  |  |  | **1.2_ Transport/binding proteins and lipoproteins** | | |
| *gutA* | 2.8 |  |  | probable glucitol transport protein GutA | |
| *citM* | 2.4 |  |  | magnesium citrate secondary transporter CitM | |
| *glvC* | 2.5 |  |  | phosphotransferase system (PTS) maltose-specificenzyme IICB component GlvC | |
| *appF* | 1.5 |  |  | oligopeptide transport ATP-binding protein AppF | |
| *oppA* | 1.5 |  |  | oligopeptide ABC transporter (binding protein) OppA | |
| *oppD* | 1.5 |  |  | oligopeptide ABC transporter (ATP-binding protein) OppD | |
| *oppF* | 1.6 |  |  | oligopeptide ABC transporter (ATP-binding protein) OppF | |
| *cysP* | 1.8 |  |  | sulfate permease CysP | |
| *ebrB* | 1.8 |  |  | multidrug resistance protein EbrB | |
| *araQ* | 1.9 |  |  | L-arabinose transport system permease protein AraQ | |
| *araP* | 2.4 |  |  | L-arabinose transport system permease protein AraP | |
| *araN* | 2.2 |  |  | probable arabinose-binding protein precursor AraN | |
| *amyC* | 1.8 |  |  | maltose transport protein AmyC | |
| *mscL* | 1.8 |  |  | Large conductance mechanosensitive channel protein MscL | |
| licA | 1.5 |  |  | phosphotransferase system (PTS) lichenan specific enzyme IIA component LicA | |
| iolF | 2.1 |  |  | inositol transport protein IolF | |
| rocE | 4.0 |  |  | amino acid permease RocE | |
| ykoE | -1.5 |  |  | thiamine ABC transporter (membrane protein), thiamine uptake | |
|  |  |  |  |  | |
|  |  | **2_Intermediary metabolism** | | | |
|  |  |  | **2.1_ Metabolism of carbohydrates and related molecules** | | |
| *acoL* | 1.5 |  |  | acetoin dehydrogenase E3 component (dihydrolipoamide dehydrogenase) AcoL | |
| *ald* | -2.4 |  |  | alanine dehydrogenase Ald | |
| *ansA* | 1.6 |  |  | L-asparaginase AnsA | |
| *araB* | 1.7 |  |  | L-ribulokinase AraB | |
| *araD* | 1.8 |  |  | L-ribulose-5-phosphate 4-epimerase AraD | |
| *araL* | 2.6 |  |  | arabinose operon protein L (AraL) | |
| *araM* | 2.3 |  |  | arabinose operon protein M (AraM) | |
| *citB* | 1.7 |  |  | aconitate hydratase CitB | |
| *citZ* | 2.3 |  |  | citrate synthase II CitZ | |
| *galE1* | 1.6 |  |  | UDP-glucose 4-epimerase GalE1 | |
| *galK1* | 5.3 |  |  | galactokinase GalK1 | |
| *galT1* | 4.2 |  |  | galactose-1-phosphate uridyltransferase GalT1 | |
| *gapB* | 1.6 |  |  | glyceraldehyde-3-phosphate dehydrogenase GapB | |
| *glpK* | 1.5 |  |  | glycerol kinase (ATP:glycerol 3-phosphotransferase) (Glycerokinase) GlpK | |
| *glvA* | 5.2 |  |  | maltose-6'-phosphate glucosid GlvA | |
| *iolB* | 2.7 |  |  | inositol utilization protein B (IolB) | |
| *iolC* | 4.2 |  |  | inositol utilization protein C (IolC) | |
| *iolD* | 4.2 |  |  | inositol utilization protein D (IolD) | |
| *iolE* | 2.8 |  |  | inositol utilization protein E (IolE) | |
| *iolG* | 2.5 |  |  | myo-inositol 2-dehydrogenase IolG | |
| *iolI* | 2 |  |  | inositol utilization protein I (IolI) | |
| *iolS* | 1.7 |  |  | inositol utilization protein S (IolS) | |
| *lacE* | 1.6 |  |  | phosphotransferase system (PTS) lichenan-specific enzyme IIC component LacE | |
| *lacF* | 1.8 |  |  | phosphotransferase system cellobiose-specific component LacF | |
| *licH* | 1.6 |  |  | 6-phospho-beta-glucosidase LicH | |
| *mdh* | 1.9 |  |  | malate dehydrogenase Mdh | |
| *odhB* | 2 |  |  | dihydrolipoyllysine-residue succinyltransferase component of 2-oxoglutarate dehydrogenase complex OdhB | |
| *pdhC* | 1.5 |  |  | pyruvate dehydrogenase (dihydrolipoamide acetyltransferase E2 subunit) PdhC | |
| *pgi* | 1.5 |  |  | glucose-6-phosphate isomerase Pgi | |
| *pgk* | 2.4 |  |  | phosphoglycerate kinase Pgk | |
| *pgm2* | 1.8 |  |  | phosphoglyceromutase Pgm2... | |
| *rpe* | 1.5 |  |  | ribulose-5-phosphate 3-epimerase Rpe | |
| *sdhB* | 1.5 |  |  | succinate dehydrogenase (iron-sulfur protein) SdhB | |
| *sucC* | 1.9 |  |  | succinyl-CoA synthetase (beta subunit) SucC | |
| *sucD* | 1.7 |  |  | succinyl-CoA synthetase (alpha subunit) SucD | |
| *ydjE* | 1.6 |  |  | fructokinase homolog YdjE | |
|  |  |  | **2.2_ Metabolism of amino acids and related molecules** | | |
| *gcvPB* | 1.6 |  |  | glycine decarboxylase (subunit 2) (glycine cleavage system protein P) GcvPB | |
| *gcvT* | 1.8 |  |  | aminomethyltransferase (glycine cleavage system protein T) GcvT | |
| *gudB* | 1.5 |  |  | NAD-specific glutamate dehydrogenase GudB | |
| *iolA* | 2.7 |  |  | methylmalonate-semialdehyde dehydrogenase IolA | |
| *kbl* | 2.2 |  |  | 2-amino-3-ketobutyrate coenzyme A ligase Kbl... | |
| *proA* | -1.6 |  |  | gamma-glutamyl phosphate reductase ProA | |
| *rocD* | 6.5 |  |  | ornithine aminotransferase RocD | |
| *rocF* | 5.4 |  |  | arginase RocF | |
| *tdh* | 3.2 |  |  | L-threonine 3-dehydrogenase Tdh | |
| *thrB* | -1.5 |  |  | homoserine kinase ThrB | |
|  |  |  | **2.5_ Metabolism of coenzymes and prosthetic groups** | | |
| *pabC* | 1.7 |  |  | aminodeoxychorismate lyase PabC | |
| *hepT* | 2.0 |  |  | heptaprenyl diphosphate synthase component II HepT | |
| *folC* | 1.7 |  |  | folyl-polyglutamate synthetase FolC | |
| *ywkE* | 1.6 |  |  | hemK protein homolog YwkE | |
|  |  |  | **2.4_ Metabolism of lipids** | | |
| *scoB* | 1.6 |  |  | succinyl CoA:3-oxoacid CoA-transferase (subunit B) ScoB | |
| *yngG* | -1.5 |  |  | hydroxymethylglutaryl-CoA lyase homolog YngG | |
| *bkdB* | 1.9 |  |  | branched-chain alpha-keto acid dehydrogenase E2 subunit (lipoamide acyltransferase) BkdB | |
| *bkdAA* | 1.7 |  |  | branched-chain alpha-keto acid dehydrogenase E1 subunit (2-oxoisovalerate dehydrogenase alpha) bBkdAA | |
| *bcd* | 1.8 |  |  | leucine dehydrogenase Bcd | |
|  |  |  | **2.3_ Metabolism of nucleotides and nucleic acids** | | |
| *nin* | 1.5 |  |  | inhibitor of the DNA degrading activity of NucA (competence) Nin | |
| *pyrF* | -1.6 |  |  | orotidine 5'-phosphate decarboxylase PyrF | |
| *pyrH* | 1.5 |  |  | uridylate kinase PyrH | |
| *cdd* | 1.7 |  |  | cytidine deaminase Cdd | |
|  |  |  |  |  | |
|  |  | **4_Other functions** | | | |
|  |  |  | **4.1_ Adaptation to atypical conditions** | | |
| *ykrL* | 1.5 |  |  | protease htpx homolog YkrL | |
| *degR* | 1.5 |  |  | regulatory protein DegR | |
| *grpE* | 1.5 |  |  | heat-shock protein GrpE | |
| *ytxG* | 1.5 |  |  | general stress protein | |
| *yqjL* | 1.5 |  |  | general stress protein, putative hydrolase involved in oxidative stress resistance | |
| *yqeZ* | 2.0 |  |  | seine protease, resistence protein (against sublancin) | |
|  |  |  | **4.2_ Detoxification** | | |
| *yceD* | 1.7 |  |  | general stress protein, similar to tellurium resistance protein | |
| *yceE* | 1.8 |  |  | general stress protein, similar to tellurium resistance protein | |
| *yceF* | 1.7 |  |  | general stress protein, similar to tellurium resistance protein | |
| *yfhL* | 1.5 |  |  | general stress protein, resistence protein (against toxic peptide SdpC) | |
|  |  |  | **4.6_ Miscellaneous** | | |
| *ctaG* | 1.5 |  |  | formation of functional cytochrome C-oxidase (caa3) | |
| *era* | 2.2 |  |  | GTP-binding protein Era | |
| *yurV* | 1.7 |  |  | iron-sulfur cofactor synthesis protein nifU homolog YurV | |
|  |  |  | **4.4_ Phage-related functions** | | |
| *xhlA* | 1.6 |  |  | | phage-like element PBSX protein XhlA |
|  |  |  | **4.3_ Antibiotic production** | | |
| *baeE* | 1.6 |  |  | malonyl-CoA-[acyl-carrier protein] transacylase BaeE | |
| *baeI* | 2.2 |  |  | enoyl-CoA-hydratase BaeI | |
| *baeL* | 1.9 |  |  | polyketide synthase BaeL | |
| *baeN* | 1.5 |  |  | hybrid NRPS/PKS BaeN | |
| *baeR* | 2.3 |  |  | polyketide synthase BaeR | |
| *difJ* | 2.0 |  |  | modular polyketide synthase of type I DifJ | |
| *difI* | 1.7 |  |  | modular polyketide synthase of type I DifI | |
| *difG* | 2.0 |  |  | modular polyketide synthase of type I DifG | |
| *difF* | 2.4 |  |  | modular polyketide synthase of type I DifF | |
| *mlnH* | 1.5 |  |  | polyketide synthase of type I MlnH | |
| *fenE* | 1.5 |  |  | fengycin synthetase FenE | |
| *srfAD* | 1.9 |  |  | surfactin synthetase D SrfAD | |
| *srfAC* | 1.7 |  |  | surfactin synthetase C SrfAC | |
|  |  |  |  |  | |
|  |  | **3_Information pathways** | | | |
|  |  |  | **3.3_ DNA recombination** | | |
| *recA* | 1.6 |  |  | multifunctional SOS repair regulator RecA | |
|  |  |  | **3.1_ DNA replication** | | |
| *priA* | 1.5 |  |  | primosomal protein N' PriA | |
| *ssb* | 1.6 |  |  | single-strand DNA-binding protein (Helix-destabilizing protein) Ssb | |
| *yneE* | -1.5 |  |  | sporulation protein, inhibits DNA replication, control of chromosome copy number | |
|  |  |  | **3.8_ Protein modification** | | |
| *map* | 3.1 |  |  | methionine aminopeptidase Map | |
| *prpC* | 1.7 |  |  | protein phosphatase PrpC | |
|  |  |  | **3.7_ Protein synthesis** | | |
| *alaS* | -1.5 |  |  | alanyl-tRNA synthetase AlaS | |
| *fusA* | 2.2 |  |  | elongation factor G FusA | |
| *tufA* | 1.5 |  |  | elongation factor Tu TufA | |
| *lepA* | 1.5 |  |  | GTP-binding protein LepA | |
| *infA* | 2.0 |  |  | translation initiation factor IF-I InfA | |
| *infB* | 1.6 |  |  | initiation factor (IF-2) InfB | |
| *infC* | 1.8 |  |  | initiation factor IF-3 InfC | |
| *rplA* | 1.7 |  |  | ribosomal protein L1 (BL1) RplA | |
| *rplJ* | 2.0 |  |  | ribosomal proteinL10 (BL5) RplJ | |
| *rplD* | 1.8 |  |  | ribosomal protein L4 RplD | |
| *rpsM* | 1.6 |  |  | ribosomal protein S13 RpsM | |
| *rpsK* | 1.6 |  |  | ribosomal protein S11 (BS11) RpsK | |
| *rplM* | 1.8 |  |  | ribosomal protein L13 RplM | |
| *rpsI* | 1.7 |  |  | ribosomal protein S9 RpsI | |
| *rpsO* | 1.6 |  |  | ribosomal protein S15 (BS18) RpsO | |
| *rpmGA* | 1.7 |  |  | 50S ribosomal protein L33 type I RpmGA | |
| *rpsU* | 3.1 |  |  | ribosomal protein S21 RpsU | |
| *rpmA* | 1.6 |  |  | 50S ribosomal protein L27 (BL30) (BL24) RpmA | |
| *rplU* | 2.0 |  |  | 50S ribosomal protein L21 (BL20) RplU | |
| *rpsR* | 2.1 |  |  | ribosomal protein S18 RpsR | |
|  |  |  | **3.6_ RNA modification** | | |
| *trmU* | 1.5 |  |  | tRNA (5-methylaminomethyl-2-thiouridylate) methyltransferase TrmU | |
|  |  |  | **3.5_ RNA synthesis** | | |
| *ydcE* | 1.5 |  |  | RNase EndoA, MazF family toxin, cleaves cellular mRNAs at specific, but frequently occuring sites | |
| *yjbH* | 1.5 |  |  | adaptor protein for ClpX-ClpP-catalyzed Spx degradation | |
| *ykqC* | 1.6 |  |  | RNase J1, RNA processing, subject to Clp-dependent proteolysis upon glucose starvation | |
| *ymdA* | 1.6 |  |  | RNase Y, 5' end sensitive endoribonuclease, involved in the degradation/processing of mRNA | |
| *rpoC* | 1.9 |  |  | RNA polymerase (beta subunit) RpoC | |
| *rpoA* | 2.0 |  |  | RNA polymerase (alpha subunit) RpoA | |
| *sigW* | 2.4 |  |  | rNA polymerase ECF-type sigma factor SigW | |
| *yjbD* | 1.5 |  |  | Transcriptional regulator Spx, involved in regulation of many genes. | |
| *glvR* | 4.4 |  |  | HTH-type transcriptional regulator GlvR | |
| *perR* | 2.2 |  |  | peroxide operon regulator PerR | |
| *glpP* | 1.8 |  |  | glycerol uptake operon antiterminator regulatoryprotein GlpP | |
| *hpr* | 1.5 |  |  | protease production regulatory protein Hpr | |
| *fapR* | 1.5 |  |  | transcription factor (Fatty acid and phospholipid biosynthesis regulator) FapR | |
| *glnR* | 1.8 |  |  | glutamine synthetase transcription repressor GlnR | |
| *hrcA* | 1.9 |  |  | heat-inducible transcription repressor HrcA | |
| *phoP* | 1.9 |  |  | alkaline phosphatase synthesis transcriptional regulatory protein PhoP | |
| *spoIIID* | -1.5 |  |  | stage III sporulation protein D (SpoIIID) | |
| *yqzJ* | 1.5 |  |  | ribosome-nascent chain sensor of membrane protein biogenesis | |

Remark: FCH: fold change. All genes listed were with a q value of no more than 0.01 and a fold change of no less than 1.5. Those with a minus value of fold change are the genes which were down-regulated by the root exudates.

### SM Table 2: The genes of FZB42 with putative function or encoding hypothetical protein whose transcriptions were significantly altered in response to maize root exudates at OD3.0 (Refer to experiment “Response to RE”: E-MEXP-3421).

| ***Gene*** | ***FCH*** |  |  | ***Product*** |
| --- | --- | --- | --- | --- |
|  |  | **1_cell envelope and cellular processes** | | |
|  |  |  | **1.2_ Transport/binding proteins and lipoproteins** | |
| *ykqB* | 1.6 |  |  | conserved hypothetical protein YkqB |
| *yqeW* | -1.5 |  |  | conserved hypothetical protein YqeW |
| *yyaJ* | -1.6 |  |  | conserved hypothetical protein YyaJ |
| *ytrE* | -1.5 |  |  | hypothetical ABC transporter ATP-binding proteinYtrE |
| *yufN* | 1.7 |  |  | hypothetical lipoprotein YufN |
| *RBAM00714* | -1.5 |  |  | putative ABC transporter (ATP-binding protein) RBAM00714 |
| *RBAM03581* | -1.5 |  |  | putative ABC transporter ATP-binding protein RBAM03581 |
| *RBAM00715* | -1.7 |  |  | putative ABC transporter permease RBAM00715 |
| *yknZ* | 1.7 |  |  | putative ABC transporter permease YknZ |
| *ytnA* | 1.9 |  |  | putative amino acid permease YtnA |
| *ytmK* | 1.6 |  |  | putative amino-acid ABC transporter (extracellular binding protein) YtmK |
| *cimH* | 1.6 |  |  | putative citrate/malate transporter CimH |
| *ydjK* | 2.3 |  |  | putative sugar transporter YdjK |
|  |  |  | **1.3_ Sensors (signal transduction)** | |
| *mrsK2* | 1.5 |  |  | putative sensor histidine kinase MrsK2 |
|  |  |  | **1.7_ Cell division** | |
| *yacA* | 1.5 |  |  | conserved hypothetical protein YacA |
|  |  | **2_intermediary metabolism** | | |
|  |  |  | **2.1_ Metabolism of carbohydrates and related molecules** | |
| *pgm1* | 2.4 |  |  | predicted phosphatase/phosphohexomutase Pgm1 |
| *lacG* | 2.7 |  |  | putative 6-phospho-beta-galactosidase LacG |
| *ycsN* | 1.6 |  |  | putative aryl-alcohol dehydrogenase YcsN |
| *ydjL* | 1.5 |  |  | putative dehydrogenase YdjL |
| *epsE* | 1.5 |  |  | putative exopolysaccharide biosynthesis protein EspE |
| *RBAM02462* | -1.5 |  |  | putative polysaccharide deacetylase RBAM02462 |
|  |  |  | **2.2_ Metabolism of amino acids and related molecules** | |
| *ymfH* | -1.5 |  |  | conserved hypothetical protein YmfH |
| *yisK* | 1.6 |  |  | putative 5-oxo-1,2,5-tricarboxilic-3-penten aciddecarboxylase YisK |
| *cysC* | 1.5 |  |  | putative adenylyl-sulfate kinase CysC |
| *yurP* | -1.9 |  |  | putative glutamine-fructose-6-phosphate transaminase YurP |
| *yurL* | -1.5 |  |  | putative sugar kinase YurL |
|  |  |  | **2.3_ Metabolism of nucleotides and nucleic acids** | |
| *yabR* | 1.7 |  |  | putative polyribonucleotide nucleotidyltransferase YabR |
|  |  |  | **2.4_ Metabolism of lipids** | |
| *ycsD* | 1.8 |  |  | conserved hypothetical protein YcsD |
| *yusL* | 1.6 |  |  | putative 3-hydroxyacyl-CoA dehydrogenase YusL |
| *ydbM* | 1.5 |  |  | putative butyryl-CoA dehydrogenase YdbM |
| *ptb* | 1.7 |  |  | putative phosphate butyryltransferase Ptb |
|  |  |  | **2.7_Metabolism of sulfur** | |
| *yvgQ* | 1.5 |  |  | putative sulfite reductase YvgQ |
|  |  | **3_information pathways** | | |
|  |  |  | **3.3_ DNA recombination** | |
| *yrrC* | -1.5 |  |  | conserved hypothetical protein YrrC |
|  |  |  | **3.5_ RNA synthesis** | |
| *ydeB* | 2.9 |  |  | conserved hypothetical protein YdeB |
| *yvyD* | 1.8 |  |  | conserved hypothetical protein YvyD |
| *ybbM* | 3.2 |  |  | predicted transmembrane transcriptional regulator (anti-sigma W factor) YbbM |
| *yybE* | -1.7 |  |  | putative HTH-type transcriptional regulator YybE |
| *lacR* | 1.5 |  |  | putative lactose phosphotransferase system repressor protein LacR |
| *RBAM00542* | -1.7 |  |  | putative transcriptional regulator (GntR family)RBAM00542 |
|  |  |  | **3.7_ Protein synthesis** | |
| *ybxF* | 2.0 |  |  | conserved hypothetical protein YbxF |
|  |  |  | **3.8_ Protein modification** | |
| *yxaL* | 1.5 |  |  | conserved hypothetical protein YxaL |
|  |  | **4_other functions** | | |
|  |  |  | **4.2_ Detoxification** | |
| *yceH* | 1.7 |  |  | putative toxic anion resistance protein YceH |
|  |  |  | **4.3_ Antibiotic production** | |
| *dfnY* | 1.7 |  |  | hypothetical protein DifY |
|  |  |  | **4.6_ Miscellaneous** | |
| *veg* | 2.8 |  |  | conserved hypothetical proteinVeg |

Remark: FCH: fold change. All genes listed were with a q value of no more than 0.01 and a fold change of no less than 1.5. Those with a minus value of fold change are the genes which were down-regulated by the root exudates.

### SM Table 3: The genes of FZB42 with unknown function whose transcriptions were significantly altered in response to maize root exudates at OD3.0 (Refer to experiment “Response to RE”: E-MEXP-3421).

| ***Gene*** | ***FCH*** | ***Description*** |
| --- | --- | --- |
| *engC* | 1.8 | Similar to unknown proteins from B. subtilis |
| *RBAM00434* | 2.5 | No similarity |
| *RBAM00435* | 1.7 | Similar to unknown proteins from other organisms |
| *RBAM00520* | 2.3 | Similar to unknown proteins from other organisms |
| *RBAM00685* | 1.5 | No similarity |
| *RBAM01042* | 1.6 | Similar to unknown proteins from other organisms |
| *RBAM01125* | -1.5 | Similar to unknown proteins from other organisms |
| *RBAM01763* | -2 | Similar to unknown proteins from other organisms |
| *RBAM01835* | -1.6 | No similarity |
| *RBAM01886* | 1.5 | No similarity |
| *RBAM01923* | -1.5 | No similarity |
| *RBAM01955* | 1.5 | Similar to unknown proteins from other organisms |
| *RBAM02215* | 1.6 | Similar to unknown proteins from other organisms |
| *RBAM02992* | 1.6 | No similarity |
| *RBAM03094* | 1.7 | Similar to unknown proteins from other organisms |
| *RBAM03224* | -1.6 | No similarity |
| *RBAM03268* | 1.9 | Similar to unknown proteins from other organisms |
| *RBAM03561* | 1.8 | No similarity |
| *RBAM03844* | -1.8 | No similarity |
| *RBAM03862* | -1.6 | No similarity |
| *yaaA* | 1.9 | Similar to unknown proteins from B. subtilis |
| *yaaR* | 1.6 | Similar to unknown proteins from B. subtilis |
| *ybbR* | 1.5 | Similar to unknown proteins from B. subtilis |
| *ybfQ* | 1.5 | Similar to unknown proteins from B. subtilis |
| *ycgB* | 1.5 | Similar to unknown proteins from B. subtilis |
| *ydcD* | 2.2 | Similar to unknown proteins from B. subtilis |
| *ydjI* | 1.5 | Similar to unknown proteins from B. subtilis |
| *yebC* | 1.5 | Similar to unknown proteins from B. subtilis |
| *yfhH* | 1.6 | Similar to unknown proteins from B. subtilis |
| *yfiT* | 1.5 | Similar to unknown proteins from B. subtilis |
| *yfjT* | -1.8 | Similar to unknown proteins from B. subtilis |
| *yflN* | 1.7 | Similar to unknown proteins from B. subtilis |
| *yheA* | 1.7 | Similar to unknown proteins from B. subtilis |
| *yhjN* | 1.5 | Similar to unknown proteins from B. subtilis |
| *yjlC* | 1.5 | Similar to unknown proteins from B. subtilis |
| *ykyA* | 1.7 | Similar to unknown proteins from B. subtilis |
| *ylbK* | -1.6 | Similar to unknown proteins from B. subtilis |
| *ylbN* | 1.6 | Similar to unknown proteins from B. subtilis |
| *yllB* | 2.1 | Similar to unknown proteins from B. subtilis |
| *ylqC* | 1.8 | Similar to unknown proteins from B. subtilis |
| *ylqD* | 1.6 | Similar to unknown proteins from B. subtilis |
| *ymcB* | 2.1 | Similar to unknown proteins from B. subtilis |
| *yngL* | 2 | Similar to unknown proteins from B. subtilis |
| *yodA* | 1.8 | Similar to unknown proteins from B. subtilis |
| *ypbS* | 1.5 | Similar to unknown proteins from B. subtilis |
| *ypeP* | 1.5 | Similar to unknown proteins from B. subtilis |
| *ypiB* | 2 | Similar to unknown proteins from B. subtilis |
| *ypmA* | 1.6 | Similar to unknown proteins from B. subtilis |
| *ypmP* | 2.2 | Similar to unknown proteins from B. subtilis |
| *yppF* | 1.5 | Similar to unknown proteins from B. subtilis |
| *yqeY* | 2.5 | Similar to unknown proteins from B. subtilis |
| *yqhY* | 1.5 | Similar to unknown proteins from B. subtilis |
| *yqkC* | 1.8 | Similar to unknown proteins from B. subtilis |
| *yqxD* | 1.5 | Similar to unknown proteins from B. subtilis |
| *yqzC* | 1.7 | Similar to unknown proteins from B. subtilis |
| *yrdA* | 1.8 | Similar to unknown proteins from B. subtilis |
| *yrkF* | 1.9 | Similar to unknown proteins from B. subtilis |
| *yrrK* | 1.6 | Similar to unknown proteins from B. subtilis |
| *yrzL* | 1.6 | Similar to unknown proteins from B. subtilis |
| *yubD* | 2.1 | Similar to unknown proteins from B. subtilis |
| *yukE* | 1.7 | Similar to unknown proteins from B. subtilis |
| *yviA* | 1.5 | Similar to unknown proteins from B. subtilis |
| *yvqI* | 1.5 | Similar to unknown proteins from B. subtilis |
| *ywcI* | -4 | Similar to unknown proteins from B. subtilis |
| *ywlA* | 1.6 | Similar to unknown proteins from B. subtilis |
| *ywqB* | -1.6 | Similar to unknown proteins from B. subtilis |
| *yxjC* | 1.9 | Similar to unknown proteins from B. subtilis |
| *yxxF* | -1.5 | Similar to unknown proteins from B. subtilis |
| *yydA* | -1.6 | Similar to unknown proteins from B. subtilis |

Remark: FCH: fold change. All genes listed were those with a q value of no more than 0.01 and a fold change of no less than 1.5. Those with a minus value of fold change are the genes which were down-regulated by the root exudates.

### SM Table 4: The primers used for real-time PCR

| **Oligo Name** | **Oligo Sequence (5‘ – 3‘)** | Length (nt) |
| --- | --- | --- |
| baeI1_fr | CACTTGGTGACGCCGTTTC | 19 |
| dfnJ1_fr | GTCGGCATGGGAGAGGAA | 18 |
| glvA1_fr | CGGATGATATGGTGAAAAAATCAA | 24 |
| hag1_fr | GCTGAGGGTGCATTAAACGAA | 21 |
| iolA1_fr | AGCGCGTGCAAGCGTTA | 17 |
| iolD1_fr | AGCAGGTGGAGCAGGAATACA | 21 |
| ptb1_fr | GGGAACCCTATGCCGAAAG | 19 |
| sigW1_fr | AGCAGAAGGGCTGACGATGT | 20 |
| ydbM1_fr | GCCTGAACGGACCGATTAAA | 20 |
| yfjT1_fr | GACCCTGAATCAACGGACGTT | 21 |
|  |  |  |
| baeI2_rev | CGTGCATGATTAACTCCTTCTCA | 23 |
| dfnJ2_rev | GGGCCGGTTTATGATAGACTTG | 22 |
| glvA2_rev | TTCCCGCCCTTCCATGA | 17 |
| hag2_rev | CGTTAGCCGCTTGTGTAGCA | 20 |
| iolA2_rev | CTTCAAGGTGGGCGTCATTT | 20 |
| iolD2_rev | GCGGGACACGGGCTTTA | 17 |
| ptb2_rev | CGCCTCCATTTTCGGATTAA | 20 |
| sigW2_rev | ACGGCGTCTTCAGGGAGAA | 19 |
| ydbM2_rev | GCTCCATTTCCCCGATACG | 19 |
| yfjT2_rev | GACCCTGAATCAACGGACGTT | 21 |
