# Supplementary material for: Transcriptomic profiling of Bacillus amyloliquefaciens FZB42 in response to maize root exudates
Source: BMC Microbiol. 2012 Jun 21;12:116. doi: 10.1186/1471-2180-12-116 (PMC3438084; doi:10.1186/1471-2180-12-116)
Supplement: Additional file 2 — Table S5. Differentially expressed genes of FZB42 in response to IE compared with those to RE (Refer to experiment “IE <> RE”: E-MEXP-3553). The genes highlighted were those with a q value of ≤0.01. (DOC 33 kb) [file 1471-2180-12-116-S2.doc]

## Table 5: Differentially expressed genes of FZB42 in response to IE compared with those to RE (Refer to experiment “IE <> RE”: E-MEXP-3553). The genes highlighted were those with a q value of ≤0.01.

| ***Gene*** | ***Product*** | ***q value*** | ***FCH***  ***wt+IE<>wt+RE*** |
| --- | --- | --- | --- |
| *ggt* | gamma-glutamyltranspeptidase Ggt | 0.00 | 2.2 |
| *RBAM00438* | hypothetical protein RBAM00438 | 0.00 | 1.5 |
| *nprE* | bacillolysin precursor NprE | 0.01 | 1.5 |
| *clpP* | ATP-dependent Clp protease proteolytic subunit ClpP | 0.00 | 1.5 |
| *ywcE* | hypothetical protein YwcE | 0.02 | 1.5 |
| *ydjO* | hypothetical protein YdjO | 0.02 | 1.7 |
| *RBAM03284* | ribonuclease precursor (Barnase) RBAM03284 | 0.02 | 1.5 |
| *bglS* | endo-beta-1,3-1,4 glucanase BglS | 0.05 | 1.6 |
| *RBAM00226* | hypothetical protein RBAM00226 | 0.04 | -1.6 |

Abbreviations used here represents: FCH: fold change; wt: FZB42 wild type; IE: the “interaction exudates”; RE: the common “root exudates”; +: in the presence of root exudates or soil extract
