# Supplementary material for: Transcriptomic profiling of Bacillus amyloliquefaciens FZB42 in response to maize root exudates
Source: BMC Microbiol. 2012 Jun 21;12:116. doi: 10.1186/1471-2180-12-116 (PMC3438084; doi:10.1186/1471-2180-12-116)
Supplement: Additional file 3 — Table S6. Microarray experimental design and data bank accession. (DOC 40 kb) [file 1471-2180-12-116-S3.doc]

## Table 6: Microarray experimental design and data bank accession.

| **Experiment**  **name** | **Treatment <> Control** | **Nr. of biological replicates** | **ArrayExpress accession** |
| --- | --- | --- | --- |
| Pilot experiment | OD1.0+RE0.25 <> OD1.0-RE | 1 | E-MEXP-3554 |
|  | OD1.0+RE0.5 <> OD1.0-RE | 1 |  |
|  | OD1.0+RE1.0 <> OD1.0-RE | 1 |  |
|  | OD3.0+RE0.25 <> OD3.0-RE | 1 |  |
|  | OD3.0+RE0.5 <> OD3.0-RE | 1 |  |
|  | OD3.0+RE1.0 <> OD3.0-RE | 1 |  |
|  |  |  |  |
| Response to RE | OD1.0+RE <> OD1.0-RE | 3 | E-MEXP-3550 |
|  | OD3.0+RE <> OD3.0-RE | 6 | E-MEXP-3421 |
|  |  |  |  |
| Response to SE | OD1.0+SE <> OD1.0-SE | 3 | E-MEXP-3551 |
|  | OD3.0+SE <> OD3.0-SE | 3 |  |
|  |  |  |  |
| IE **<>** RE | OD1.0+IE <> OD1.0+RE | 3 | E-MEXP-3553 |
|  | OD3.0+IE <> OD3.0+RE | 3 |  |

Remarks: RE: the common maize root exudates; IE: the “interaction exudates”; SE: soil extract; +: in the presence of root exudates or soil extract; -: without root exudates or soil extract; OD1.0: cells were collected when OD_600_=1.0; OD3.0: cells were collected when OD_600_=3.0. In the pilot experiment the number following RE represents the concentration (g l^-1^) of maize root applied. When not indicated, the concentration of RE or IE was 0.25 g l^-1^. In all media 10% soil extract was added, except the controls (OD1.0-SE and OD3.0-SE) of the experiment “Response to SE”, where soil extract was not added.
