# Supplementary material for: Transcriptomic profiling of Bacillus amyloliquefaciens FZB42 in response to maize root exudates
Source: BMC Microbiol. 2012 Jun 21;12:116. doi: 10.1186/1471-2180-12-116 (PMC3438084; doi:10.1186/1471-2180-12-116)
Supplement: Additional file 4 — Figure S2. Growth of FZB42 at 24°C under continuous shaking (220 rpm/min.) in medium 1 C supplemented with sterilized 10% soil extract prepared by extracting of 500 g (dry weight) compost soil with 1 L distilled water. Cells were sampled during exponential growth (OD600 = 1.0) and during transition to stationary growth phase. The time of sampling in the transition phase (O.D.600 = 3.0) is indicated by the red arrow. (DOC 32 kb) [file 1471-2180-12-116-S4.doc]

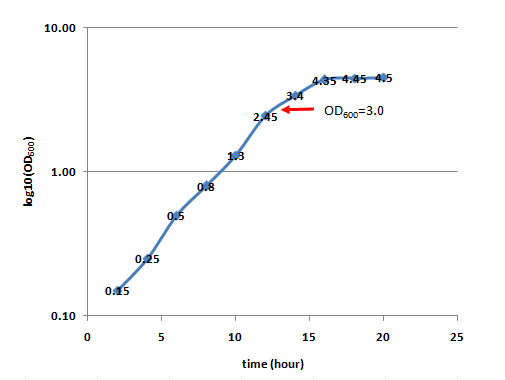


**SM Figure 1**: Growth of FZB42 at 24°C under continuous shaking (220 rpm/min.) in medium 1C supplemented with sterilized 10% soil extract prepared by extracting of 500 g (dry weight) compost soil with 1 L distilled water. Cells were sampled during exponential growth (OD_600_=1.0) and during transition to stationary growth phase. The time of sampling in the transition phase (O.D._600_=3.0) is indicated by the red arrow.
